# Standardizing deoxygenation studies in coral biology

**DOI:** 10.1038/s42003-026-11025-x

**Published:** 2026-10-19

**Authors:** Wyatt C. Million, Rachel Alderdice, Nicole J. Dilernia, Michael Kühl, Cesar O. Pacherres, Mathieu Pernice, Carly J. Randall, Alexandra Skeer, David J. Suggett, Christian R. Voolstra, Emma F. Camp

**Affiliations:** 1 Climate Change Cluster, University of Technology Sydney, Ultimo, NSW Australia; 2 Department of Biology, University of Konstanz, Konstanz, Germany; 3 Marine Biological Section, Department of Biology, University of Copenhagen, Helsingør, Denmark; 4 Australian Institute of Marine Science, Townsville, QLD Australia; 5 KAUST Coral Restoration Initiative (KCRI) and Division of Biological and Environmental Science and Engineering (BESE), King Abdullah University of Science and Technology, Thuwal, Saudi Arabia

**Keywords:** Ecophysiology, Tropical ecology, Research management, Climate-change ecology

## Abstract

Deoxygenation, the global loss of biologically available oxygen (O_2_) from the world’s oceans, presents a pervasive and intensifying threat for reef-building corals that will require coordinated efforts to understand and manage. However, recent expansion of coral deoxygenation experimentation has produced methodological gaps, limiting collective knowledge generation. Here, we show that growing divergence in this field arises primarily from differences in focal phenotypes, deoxygenation exposures, and experimental execution that together prevent biological from technical variation from being disentangled between studies. To address this, we propose a series of recommendations to establish the foundation for a future standardized framework for coral deoxygenation methodology. Such efforts are critical to establish a robust consensus of scientific data to support effective management of reef deoxygenation. Waiting decades for diverging methodologies to prompt a call for standardization may be imprudent given the urgent threat of deoxygenation and the abundant methodological divergence present in coral deoxygenation experimentation.

## Abstract (web summary)

This perspective highlights the predominant methodological barriers in coral deoxygenation research preventing cross study comparison and offers potential remedies for standardization.

## Introduction

Oxygen (O_2_) is fundamental for aerobic life and thus a prerequisite for coral reef ecosystems to form and thrive. O_2_ availability is naturally variable on tropical coral reefs^1,2^, and is influenced by O_2_ cycling between the atmosphere, neighboring water bodies, and biological components that consume, produce, and transport O_2_^3,4^. However, coastal deoxygenation^4,5^—controlled by local factors such as increased O_2_ demand, O_2_ competition, eutrophication, or water column stratification—and ocean deoxygenation^5^—arising from reduced global O_2_ solubility, increased O_2_ demand, and expanding open ocean O_2_ minimum zones^6^—can create severe and/or prolonged low-O_2_ events on tropical reefs. Depletion of ambient dissolved O_2_ concentration ([DO]) is particularly dangerous for sessile, aerobic invertebrates, including scleractinian corals, with limited mechanisms to enhance O_2_ uptake, instead relying on diffusion (mitigated by temperature, hydrodynamics, and O_2_ concentration) across the diffusion boundary layer (DBL). Although many corals host photosynthetic endosymbiotic dinoflagellates (Symbiodiniaceae) that can produce O_2_ to >250% of ambient levels, their symbiotic partner(s) compete for O_2_ at night when intratissue [DO] can reach near zero^1,7,8^. This diel O_2_ depletion is exacerbated when the [DO] of the surrounding water column is too low to meet intracellular demands. O_2_ depletion on reefs, both independently and in combination with heating, has been associated with increased coral bleaching (i.e., the breakdown of the symbiosis between corals and their photosynthetic algae)^9–11^ and mortality^12^. Because of these threats, deoxygenation is now considered a predominant stressor to reef-building corals alongside ocean warming and acidification.

As global and local drivers of deoxygenation intensify under climate change^6^, coral reefs are expected to experience longer, more frequent, and more intense low-O_2_ stress events^13^. Given its growing threat, recent efforts to characterize deoxygenation and its impacts on coral reefs have generated an influx of studies on coral deoxygenation rising from 15 to 143 from 2015 to 2025 (Supplementary Fig. 1). Alongside these studies is a growing gap in methods to assess phenotypes, design deoxygenation exposures, and execute experiments. Similar methodological divergence in other fields, e.g., ocean acidification^14^ or coral thermal bleaching^15^, resulted in decades of impactful but difficult-to-compare findings that limited collective knowledge, ultimately prompting the formation of standardized methodological frameworks^15,16^. Such standardization has yet to be proposed for ocean deoxygenation to ensure widespread efforts can be reconciled and more rapidly accelerate global understanding. Given the rapidly declining state of coral reefs around the world and the immediate and accelerating threat of ocean deoxygenation, ongoing research in the field will need to be translated to collective knowledge and incorporated into management strategies faster than ever before.

We contend that innovative research increasing our fundamental knowledge of coral responses to deoxygenation is necessary to inform a standardized experimental framework. Leveraging existing evidence, prioritizing unanswered questions, and improving transparency in methods reporting and data sharing will allow such a framework to be initialized without delay. Here, we outline how the selection of focal phenotypes, experimental deoxygenation exposures, and practical execution of experiments create discrepancies that make cross-study comparisons inappropriate, if not misleading. To overcome the methodological divergence highlighted here, we suggest best practices, when sufficient empirical evidence supports it, and identify current knowledge gaps that can inform best practices for methodological standardization. We use the recent effort to standardize methods in the coral thermal bleaching field as a case study for how such a framework can be informed by empirical evidence to provide benefits in applied and basic settings, despite potential limitations.

### Key terms in coral deoxygenation research

Hypoxia is a biological state where O_2_ supply is inadequate to sustain normal functioning within an organism^17^ which differs from deoxygenation, which is an environmental condition of low-O_2_ availability in a given system (Table 1). While often used interchangeably, hypoxia is distinctly associated with a biological state, meaning a deoxygenated environment can only be considered hypoxic if that level of low O_2_ is associated with biologically-defined hypoxia. As a result, a given O_2_ treatment may be hypoxic for one individual but not another, creating disparate ‘hypoxia’ exposure relative to species-specific hypoxia thresholds. Historically, hypoxia has been specifically associated with a detrimental physiological state, and we maintain this distinction, but acknowledge that (1) the ultimate fitness consequences of many coral phenotypic changes under deoxygenation are unresolved, and (2) thresholds for hypoxia will be dependent on the trait of interest and its direction of change. Commonly, the rate of O_2_ consumption (VO_2_ or MO_2_, used interchangeably) is used to define the upper limit of hypoxia (i.e., the onset of a hypoxic condition), but theoretically and practically, other metrics are necessary to define sublethal and lethal hypoxia thresholds. To clarify this ambiguity, we classify thresholds as aerobic (based on VO_2_), sublethal (based on biological metrics other than VO_2_), and lethal (based on mortality) (Table 1).

**Table 1:** Definitions of terms used in coral deoxygenation research (including the publications from which the current definitions were derived)

Deoxygenation	An environmental condition of low O_2_ availability relative to the natural or historical state (i.e., normoxia)^94^
Deoxygenation intensity	Concentration of dissolved O_2_ ([DO]) in a body of water below normoxia; more intense deoxygenation is associated with lower DO. Deoxygenation intensity has been described in multiple units (e.g., % air saturation, hPa, µmol O_2_ L^−1^, mL O_2_ mL^−1^, mg O_2_ mL^−1^) however conversions between should take into consideration effects on O_2_ solubility; i.e.,pressure, temperature, and salinity change. For example, % air saturation is a relative value based on atmospheric/abiotic conditions and does not describe a [DO] in the absence of temperature, pressure, and salinity compensation. (Synthesized from^5,13,95^).
Deoxygenation duration	Time a given [DO] is present within an environment or experimental condition. In situ deoxygenation can last hours to days and be frequently interrupted by reoxygenation due to photosynthesis or mixing^4,5,13^. Given the high variability of reef [DO], deoxygenation duration may be more easily characterized as the time ambient [DO] is recorded below a given threshold (*sensu* Pezner et al.^13^)
Deoxygenation severity	A composite metric to quantify deoxygenation exposure based on deoxygenation intensity and deoxygenation duration. Proposing a standardized calculation to integrate intensity and duration would be premature (see Recommendation 6) however previous studies have defined severity of deoxygenation events as the product of deoxygenation intensity and duration in hours^13^.
Normoxia	An environmental condition defined by normal O_2_ availability relative to the natural or historical state^3^
Anoxia	An environmental condition defined by the absence of O_2_^3^
Hypoxia	A biological state, where O_2_ supply is inadequate to sustain normal functioning within an organism relative to that under normoxia^17^
Hypoxia thresholds	O_2_ concentration and/or duration of exposure, at which a physiological condition deviates from homeostasis. Here, thresholds are separated into aerobic (those based on aerobic metabolism or proxies of it), sublethal (based on biological metrics other than O_2_ consumption rate (VO_2_)), and lethal (based on mortality) (Present study)
Hypoxia response curve (HRC)	Relationship between environmental O_2_ and organism VO_2_ plotted from normoxia to anoxia^25^
Oxyregulation	The ability or process of maintaining VO_2_ under declining environmental O_2_^21^
Oxyconformity	The lack of oxyregulation where VO_2_ declines in direct proportion to declining environmental O_2_^21^
Critical phenotypes	Describe O_2_ concentrations at which critical changes in rates of O_2_ consumption and therefore aerobic metabolism occur. Often these traits describe upper hypoxia thresholds. Common metrics include: ‑ P_crit_ or P_c_: the lowest concentration or saturation of O_2_ (pO_2_) at which an organism maintains a constant VO_2_, Fig. 2 ‑ P_cmax_: O_2_ concentration at maximum regulation of VO_2_ relative to a hypothetical perfect oxyconformer^21^, Fig. 2 ‑ P_mdr_: O_2_ concentration of minimum detectable regulation of VO_2_ relative to a hypothetical perfect oxyconformer^21^, Fig. 2 ‑ T_pos_: total positive VO_2_ regulation calculated as the integral of the regulation function, which is the sum of its deviation from a perfect oxyconformer from normoxia to anoxia - termed rho^21^, Fig. 2 ‑ Regulation value (R): the average VO_2_ relative to the maximum recorded VO_2_ of the organism integrated from normoxia to anoxia. Values range from 0 to 100% with 50% representing perfect oxyconformation and 100% representing perfect oxyregulation^96^ ‑ Regulation Index (RI): the proportion of the area under the HRC that is greater than a linear trend of a perfect oxyconformer. Values range from 0 (perfect oxyconformation) to 1 (perfect oxyregulation)^97^
Symptom phenotypes	Phenotypes indicative of a condition, typically reduced fitness, under deoxygenation. Includes a variety of sublethal and lethal effects of hypoxia observed in coral; these traits are also used to define sublethal and lethal hypoxia thresholds. (Present study)
Tolerance-modulating phenotypes	Observable traits or characteristics in organisms that influence or regulate tolerance. In the context of deoxygenation, they include fixed or induced morphological, behavioral, microbial or molecular traits that mitigate how corals respond to deoxygenation. Such traits have not typically been used to define hypoxia thresholds. (Present study)

Although a coral’s hypoxia response may be described in part by physiological thresholds, its complete response will include a suite of changes under deoxygenation, such as behavioral changes, changes in anaerobic metabolism, or differential gene expression^18–20^. The complexity of the coral hypoxia response creates opportunities for tradeoffs between short and long-term consequences that mitigate coral fitness under deoxygenation. Distinguishing hypoxia from deoxygenation and hypoxia thresholds from hypoxia responses not only allows environmental responses to be classified correctly, but also allows physiological responses to O_2_ to be evaluated independently. Such precision is especially important given that evolutionary and environmental history may have shaped traits, such as hypoxia responses and overall hypoxia tolerance, separately.

## Methodological divergence in coral deoxygenation experimentation

**Fig. 1:**
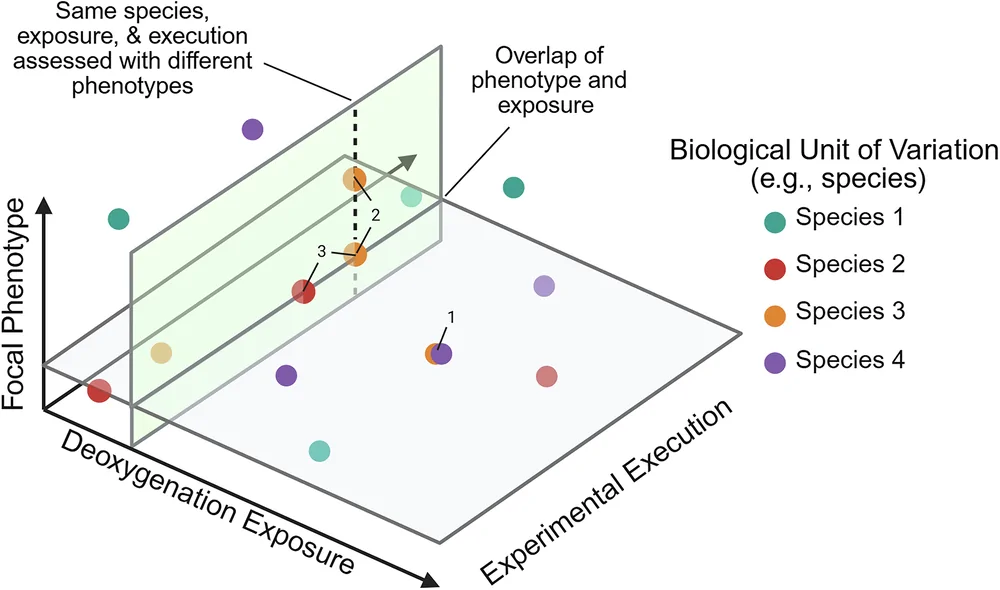
Three major components of coral deoxygenation experiments that hinder cross-study comparison. The three broad categories of methodological divergence: focal phenotype, deoxygenation exposure, and experimental execution, are represented by the x, y, and z axes. Existing studies, represented by colored points, often vary not only by the biological unit explored (e.g., species, population, life stage, or symbiont community) but also in methodological components that affect a coral’s hypoxia response. As a result, attempts to integrate datasets from multiple studies become hindered by an accumulation of differences in measured phenotypes (e.g., mortality vs. gene expression), intensity and duration of exposure to low O_2_ (e.g., intense short-term vs weak long-term), and how experiments were controlled (e.g., lighting, flow rates). Two studies occupying the same methodological space would be better positioned to attribute species-specific hypoxia responses to biological variation rather than technical variation (Comparison 1). Similarly, when the biological component is maintained across studies, comparisons of methodologies would be made possible when exposure and execution are congruent (Comparison 2). Alternatively, comparisons of biological variation across divergent methodological approaches may be possible if variation in experimental execution is able to be accounted for (Comparison 3). A call for standardization would not limit exploration of various components of deoxygenation research, but would instead create a framework for independent studies to more easily integrate their results within the broader coral deoxygenation field.

A growing body of evidence shows that focal phenotypes (e.g., aerobic respiration, behavior, mortality), experimental design (e.g., deoxygenation intensity or timing), and experimentation execution (sampling scheme or abiotic conditions) can each affect experimental outcomes in coral deoxygenation research. Currently, however, most studies simultaneously vary the measured focal phenotype, deoxygenation exposures, and experimental execution in addition to the biological components (e.g. species, population, life stage, or symbiont community) making it difficult to unequivocally attribute observed outcomes to biological variance, technical variation, or both (Fig. 1). Below, we demonstrate how these components of experimentation can impact cross-study comparisons in the absence of a standardized methodological framework and ultimately hinder the translation of individual studies into the broader knowledge generation required for urgent conservation priorities and interventions on reefs.

### Focal phenotypes

We classify the variety of phenotypes currently used to measure coral responses to reduced O_2_ availability at the organismal-level into three broad categories: critical, symptom, and tolerance-modulating. Critical phenotypes relate specifically to aerobic respiration and characterize how an organism changes its rate of O_2_ consumption under deoxygenation. Symptom phenotypes in corals define a hypoxic condition and cover sublethal and lethal effects of hypoxia. Critical and symptom phenotypes have typically been used to quantify hypoxia thresholds. Tolerance-modulating phenotypes have not traditionally been used to quantify hypoxia thresholds in corals but instead characterize a coral’s ability to adjust how it experiences or deals with deoxygenation (Table 1).

#### Critical phenotypes

**Fig. 2:**
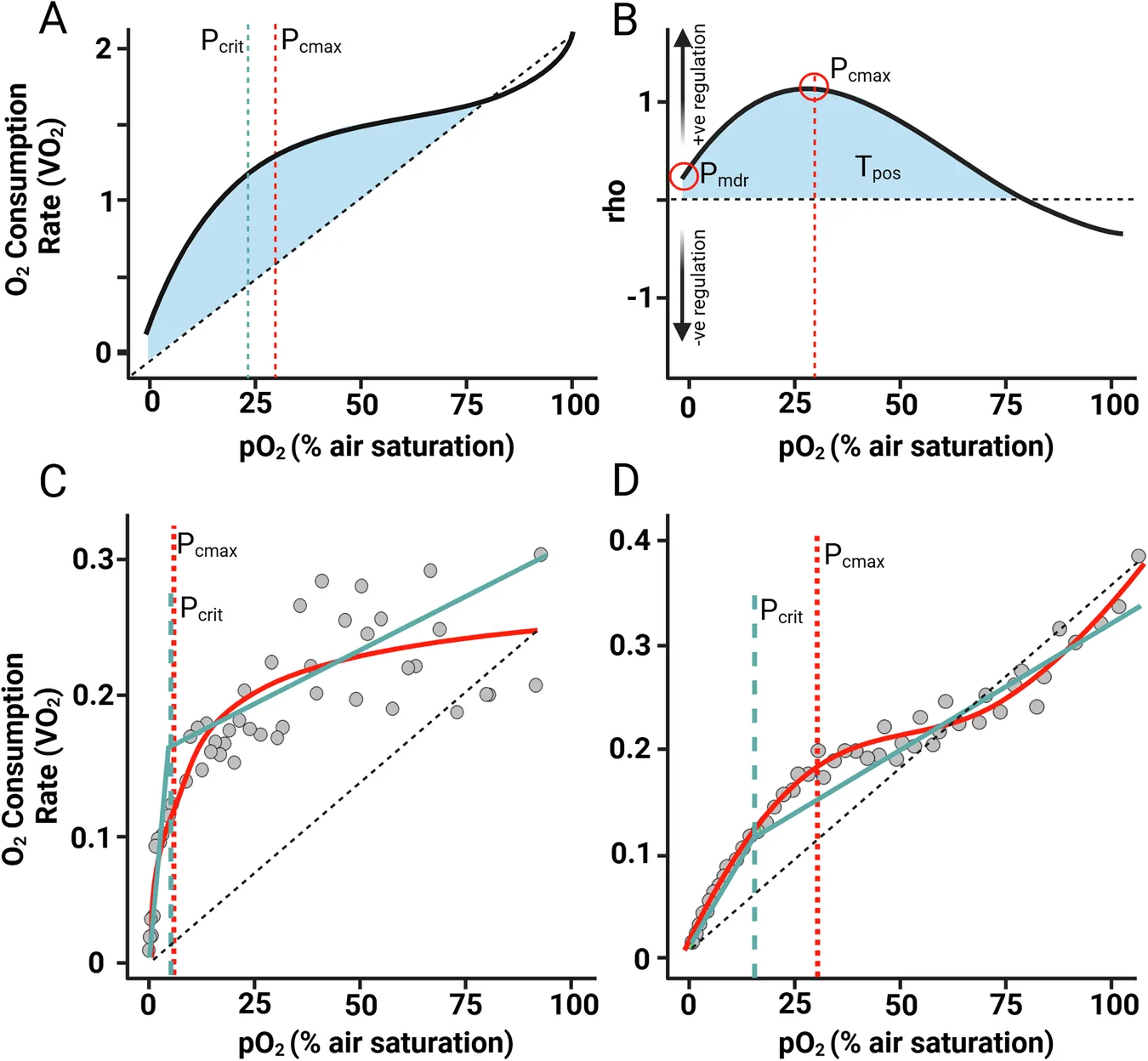
Comparison of P_crit_ and P_cmax_ across coral hypoxia response curves. Hypothetical hypoxia response curves (HRCs) with theoretical P_crit_ and P_cmax_ labeled with vertical dashed lines (**A**) and the corresponding regulation function, rho (**B**) calculated by comparing a nonlinear model fitted to the HRC with the HRC of a hypothetical perfect oxyconformer (gray dashed line). P_cmax_ and P_mdr_ are the O_2_ concentration (pO_2_) at which the maximum and minimum regulation (rho) occur as the organism approaches anoxia. The shaded region below the curve represents the integral used to calculate total positive regulation (T_pos_). The HRCs from *Porites lutea* (**C**) and *Hydnophora rigida* (**D**) reanalyzed from Hughes et al. 2022 show critical phenotypes for each species, calculated using the “broken stick” (P_crit_, green) and regulation-based methods (P_cmax_, red). The “broken stick” method (completed here with the respirometry package^98^ in R) assumes a biphasic model and determines P_crit_ at the intersection of the two linear regressions (green solid lines). The red curved lines (**C** and **D**) show the model of best fit, while the gray dashed line shows the HRC of a perfect oxyconformer.

Current critical phenotypes include traits that either describe (1) points along an O_2_ gradient where changes in VO_2_, and therefore critical metabolic shifts (e.g., P_crit_, P_c_, P_cmax_, and P_mdr_ ; Table 1) occur, or (2) the total oxyregulation of an organism (e.g., R, RI, T_pos_). Theoretically, critical phenotypes are readily comparable across aerobic organisms because they characterize changes in a conserved energetic pathway^21,22^. While all critical phenotypes can be derived from the same hypoxia response curve (HRC, Table 1, Fig. 2A), variation is introduced both when selecting and analyzing critical phenotypes - even when researchers are targeting the same conceptual physiological condition. For instance, P_c_/P_crit_ and P_cmax_ have been used to describe a limit of aerobic metabolism in corals—where regulation VO_2_ relative to ambient [DO] ceases^23^. However, multiple approaches to evaluate P_crit_ (e.g., “broken stick” and “non-linear” regression) generate divergent aerobic thresholds upon reanalysis in corals^24^, and differ from P_cmax_, which is analyzed in a regulation-based framework^21^.

To directly compare the most common critical phenotypes used to define aerobic thresholds in corals, P_crit_ and P_cmax_, we calculated both traits for two samples with diverse HRC shapes seen in coral (data from Hughes et al.^25^, see Supplementary Table 1). When a coral produces an HRC that fits the traditional shape for which P_crit_ was developed, P_crit_ and P_cmax_ are nearly identical (Fig. 2C). However, when assessing non-traditional HRC shapes like those indicative of metabolic suppression (Fig. 2D), there was a 0.82 mg O_2_ L^−1^ difference between the calculated aerobic thresholds for the same individual (30% vs 17% air saturation or 1.92 vs. 1.09 mg O_2_ L^−1^ at 28॰C, 35 ppt). To date, either P_crit_ or P_cmax_ have been used to propose aerobic hypoxia thresholds of coral species suggesting that current ranks of taxa susceptibility to deoxygenation may be unreliable. Moreover, Cobbs and Alexander^21^ reveal the majority of aquatic invertebrates analyzed did not fit the biphasic assumptions used in P_crit_ analyses suggesting similar trends may exist across taxa with a diversity of HRC shapes.

More options to classify coral hypoxia response strategies are becoming popular (P_mdr_, R, RI, and T_pos_; Table 1) and may provide more biological resolution, while also further splintering the ways researchers present critical phenotypes. For example, advances to avoid overfitting in the derivation of P_cmax_ and T_pos_ have improved confidence of critical phenotypes while diverging from historical uses^26^. Hughes et al.^25^ already identified a potential to over-estimate oxyregulation capacity when using RI rather than T_pos_ for corals showing metabolic depression. Given the diversity of coral HRCs, direct comparisons of total oxyregulation capacity between studies can also be misled by methodological variation.

#### Symptom phenotypes

**Fig. 3:**
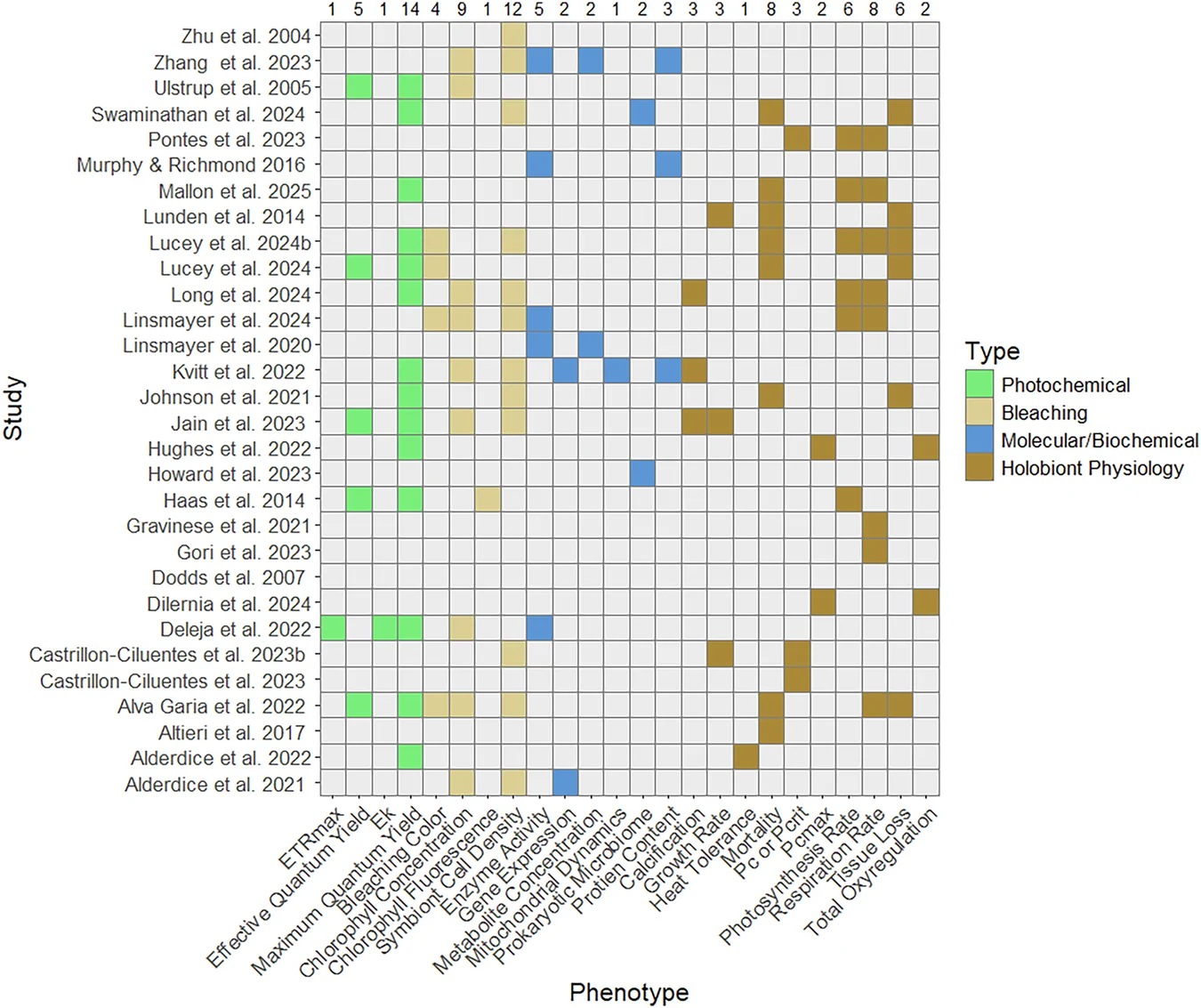
Summary of focal phenotypes targeted in coral deoxygenation experimentation. The phenotypes targeted by 30 peer-reviewed studies, which were selected for their recent (since 2004) use of experimentally manipulated O_2_ availability in adult scleractinian corals. We acknowledge this is not an exhaustive list but a representation of literature meeting this specific criterion. A filled cell means a phenotype was measured in that study, while a gray cell means it was not. Filled cells are colored by the type of phenotype explored, broadly grouped into photochemical, bleaching, molecular, and biochemical traits, and those describing holobiont physiology. Numbers above each column describe the sum of studies using a given phenotype.

A variety of physiological, chemical, and molecular symptom phenotypes have been used to classify hypoxia stress in coral. Chlorophyll concentration, symbiont cell density, and photochemical efficiency have been useful to describe degradation of Symbiodinaceae health as a proxy of holobiont health^9,10,12,18–20,27–29^. Reductions in these bleaching related traits have historically correlated to long-term fitness when induced by environmental stressors such as heat^30^ and light^11^ and in situ deoxygenation^12,31^. Measurements of photosynthesis, respiration, and growth have characterized vital holobiont processes affected by hypoxia stress^32–35^, while gene expression, metabolite concentration, and enzymatic activity have highlighted mechanisms of hypoxia response systems^19,36–38^. Finally, mortality has provided direct measures of fitness under hypoxia^12,18,39^. Although numerous, the phenotypes used to characterize coral hypoxia responses are rarely maintained across experiments. When comparing 30 studies which employed experimental deoxygenation in adult corals, we found no phenotype was measured in more than 50% of studies (Fig. 3). Moreover, the most common symptom phenotypes, maximum quantum yield (F_v_/F_m_) of the algal photosystem (14/30 studies), Symbiodiniaceae cell density (12/30), and chlorophyll *a* concentration (9/30) are specific to the algal symbionts rather than the coral animal. While the broad swath of symptom phenotypes may be necessary to characterize the full coral hypoxia response, the lack of common metrics to quantify consequences of deoxygenation in coral adds additional methodological variation, further isolating independent studies.

Quantifying the magnitude, direction, and timing of symptom phenotypes will also be critical for comparing the degree of stress experienced across studies, even when using a common set of phenotypes. For instance, Alderdice et al.^20^ showed a delayed and muted induction of hypoxia response genes (e.g., *HIFα, hsp90, LDH, PDK*) in *Acropora selago* corresponding with greater bleaching under deoxygenation relative to *Acropora tenuis* (likely *A. kenti* based on taxonomic revisions; *cf*.^40^), which showed earlier and stronger upregulation and did not bleach. In this case, differential gene expression only within the context of its timing, magnitude, and direction is capable of distinguishing between an adaptive vs. maladaptive response to deoxygenation. The fitness costs and benefits for changes in many symptom phenotypes are unclear, making the size, direction, and timing of observed changes especially important when comparing across experiments.

#### Tolerance-modulating phenotypes

Tolerance-modulating phenotypes include the morphological, behavioral, microbial, chemical, or molecular traits that mitigate how corals respond to the environment but have not typically been used to define hypoxic conditions. For example, colony morphology has been implicated in coral hypoxia tolerance, with mounding and encrusting corals tending to be more tolerant than branching corals^41–43^. Moreover, branch morphology can influence flow across a colony resulting in changes to the thickness of the DBL, altering O_2_ flux into coral tissues^44,45^. Similarly, cilia beating and polyp expansion are behaviors that can promote O_2_ uptake by disrupting the DBL and/or increasing tissue surface area^46–50^. Coral larvae show reduced substrate exploration and settlement in response to low environmental O_2_^51^. Anaerobic metabolism or metabolic suppression can be triggered by deoxygenation^19,20,36,38^ and may help meet or reduce energy demands, although downstream costs of metabolic restructuring remain unclear. Finally, the coral microbiome is an environmentally responsive holobiont “trait” known to influence performance^52^ and though unique microbiomes in low-O_2_ environments have been documented^53,54^, the relevance, robustness, and flexibility of the microbiome as a hypoxia response has only started to be explored^39,55^.

Identifying causal links between tolerance-modulating phenotypes and fitness under deoxygenation will be the next step to understanding hypoxia response systems. Mechanistic studies such as Pacherres et al.^56^ reveal the ability of cilia behavior to reduce O_2_ loss within coral tissues, linking a tolerance-modulating phenotype to buffering of intratissue hypoxia. This stands in contrast to potential correlative relationships between deoxygenation tolerance and coral morphology which may be confounded by species-specific differences and consequences of morpho-type physiology (e.g., growth rate^57,58^). Studies linking performance under deoxygenation with variation in gene expression^20^ and local adaptation^59^ offer examples of how investigating potential tolerance-modulating phenotypes in conjunction with ecologically relevant fitness metrics for coral, e.g., bleaching or mortality, can illuminate putative adaptive traits under deoxygenation. As tolerance-modulating phenotypes are investigated in greater depth, identifying approaches to best clarify when phenotypes lead to, rather than simply occur with, improvements to performance under deoxygenation will be useful for basic and applied knowledge generation.

#### Hypoxia thresholds

A hypoxia threshold characterizes the O_2_ concentration below which an organism experiences sublethal or lethal impacts^60^. Hypoxia thresholds are therefore vital for assessing and comparing taxa-specific sensitivity to deoxygenation. A high degree of standardization is required for the evaluation of hypoxia thresholds given their inherent application in cross-study comparisons. However, there are large gaps between studies in how coral hypoxia thresholds are evaluated and reported. Alva Garcia et al.^9^ exemplify this when providing a “summary of hypoxic thresholds” of coral species from seven studies that are based on four different focal phenotypes and evaluated with seven different experimental designs. To date, the experiments that have reported sublethal or lethal thresholds^9,27,28^ most frequently differ in the intensity of deoxygenation, the focal phenotype, and the cutoff for determining when a threshold has been crossed. For instance, Haas et al.^27^ reported a hypoxia threshold of 4 mg O_2_ L^−1^ for *Acropora yongei* based on significant declines in a suite of photophysiological traits. However, each evaluated trait (F_v_/F_m_ and mortality) declined at a different rate so the multivariate hypoxia threshold provided in the study creates a barrier for comparison to other hypoxia thresholds. Notably, sublethal and lethal hypoxia thresholds in other fields are based on time- or dose-dependent responses^60^, but this format has rarely been used to generate coral hypoxia thresholds^9,31^.

Phenotypes such as P_crit_ and P_cmax_ overcome the barriers for comparison outlined above by (1) exposing a coral to the same range of [DO] to produce an HRC, (2) measuring VO_2_, and (3) standardizing the cutoff for changes in VO_2_ (i.e., breakpoint of VO_2_ or point of maximum regulation). Still, the capacity of critical hypoxia thresholds as predictors of overall hypoxia tolerance in corals is still unclear. Decoupling of respiration and sublethal or lethal thresholds in fish^61^ suggests aerobic thresholds may not necessarily be a sufficient replacement for the development of standardized time- or dose-dependent hypoxia thresholds for coral.

### Deoxygenation exposure

While methods of generating deoxygenated water for experimentation may vary (see^62–64^ for practical approaches), the choice of deoxygenation exposures in coral O_2_ experimentation on symptom and tolerance-modulating phenotypes are a larger source of variation amongst existing studies. Specifically, differences in intensity, duration, and variability of deoxygenation likely contribute to the total amount of deoxygenation stress supplied and therefore require careful consideration when designing experiments. Methodologies to assess critical phenotypes bypass some of these concerns but have other unique considerations discussed below.

#### Intensity

**Fig. 4:**
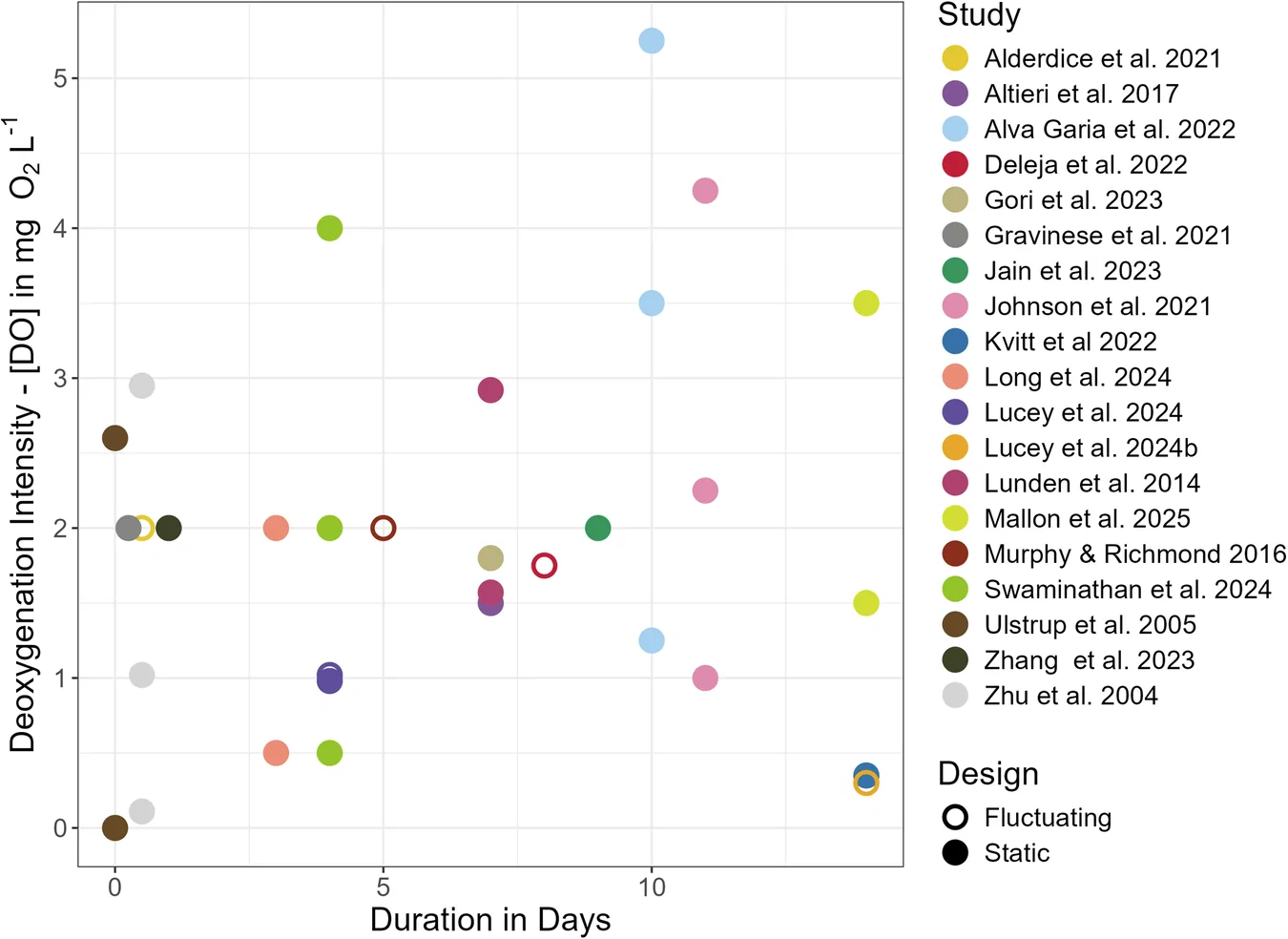
Summary of experimental deoxygenation treatments used in coral deoxygenation experimentation. Experimental deoxygenation treatments used in 21 adult coral studies surveyed in Fig. 3 did not solely evaluate critical phenotype, as they require HRC generation across a full gradient of [DO]. Studies with multiple deoxygenation treatments (excluding normoxia controls) are included with multiple colored symbols. Treatments in studies with an additional temperature-by-low O_2_ group were excluded here. Open symbols are treatments with fluctuating deoxygenation exposure (always fluctuating between treatment minimum [DO] and normoxia). Filled symbols are treatments with constant deoxygenation exposure. Deoxygenation intensity, duration, and variability represent components of the “Deoxygenation Exposure” axis within Fig. 2 that have contributed to the lack of methodological overlap between studies.

We define deoxygenation intensity as the concentration of dissolved O_2_ supplied in manipulative coral deoxygenation experiments. However, we acknowledge the experienced intensity of hypoxia stress for a coral may be dependent on a suit of factors from stress tolerance to tolerance-modulating phenotypes. A threshold of 2 mg O_2_ L^−1^, which was determined in a few species of fish and has since been erroneously generalized to marine organisms^60^, has frequently been applied in corals to evaluate symptom phenotypes (Fig. 4), despite abundant evidence that hypoxic states in many coral species occur above and below this [DO]^60,65^. Alternative approaches for selecting experimental deoxygenation intensities, e.g., those based on in situ O_2_ dynamics^12,18,28^, have resulted in a diversity of treatments (Fig. 4). However, while the experimental deoxygenation intensities used in current studies span a large range, treatments rarely overlap between studies, preventing direct comparison of hypoxia responses even when using similar focal phenotypes and species.

#### Duration and variability

The intensity, duration, and variability of [DO] corals experience in situ is influenced by forces across global/regional scales (e.g., mixing of water bodies, changes to O_2_ solubility), reef scales (e.g., O_2_ competition, flow dynamics), and at coral colony scales (e.g., respiration vs. photosynthesis and diffusion boundary layer)^1,4,5^. As a result, sporadic hypoxia events like those recorded from ambient O_2_ monitoring (e.g., ~6 days at <0.2 mg O_2_ L^−1^ in^66^) interact with the high daily variability reported on many reefs (~0 to >100% air saturation^13^) as well as rapidly changing intratissue [DO] (~0 to >250% air saturation in minutes^1^) that may exacerbate or buffer changes in ambient [DO].

To date, experimental deoxygenation exposures have ranged from hours to weeks in adult corals (Fig. 4) with exposure up to a month in coral larvae and recruits^29^. A collection of results show a time-dependent effect of deoxygenation on symptom phenotypes^10,18,31,34^ implying that similar traits measured at different times between studies will capture hypoxia responses that cannot be readily compared without accounting for duration. Variability in deoxygenation exposure has more recently been incorporated into experimental designs with day-time reoxygenation following night-time deoxygenation exposure^19,20,27,37^. While variability more closely mimics in situ O_2_ dynamics^1,7,8^, it introduces changes in the effective deoxygenation exposures, potential reoxygenation stress, and normoxia recovery periods. Constant vs. variable deoxygenation exposure has been shown to affect the timing and magnitude of coral sublethal and lethal responses to deoxygenation^31^, suggesting differences in deoxygenation variability can confound cross-study comparisons. Finally, the rate of deoxygenation (i.e., ‘ramping rate’) may present additional variation in perceived hypoxia given the potential for molecular, physiological, or behavioral plasticity to moderate tolerance under low-O_2_ conditions^37,67,68^; however, this has not been tested directly.

#### Considerations for critical phenotypes

Both closed system (CS) and intermittent flow (IF) respirometry have been used to evaluate critical phenotypes in corals. While both methods produce HRCs over a similar range of [DO], they differ in the number of points along the O_2_ gradient at which VO_2_ is measured, the exposure time to each [DO], and the rate of O_2_ depletion. CS respirometry relies on coral respiration in a sealed chamber to create a progressive O_2_ gradient such that each [DO] is experienced for only minutes^25,69,70^. Reported CS incubation times are highly variable and depend on coral respiration rate and the ratio of incubation volume to coral surface area. IF respirometry measures VO_2_ at discrete [DO] by supplying incrementally more-deoxygenated water to incubation chambers flushed after subsequent exposures, preventing buildup of metabolic byproducts. Uses of IF respirometry in corals measure VO_2_ during hours-long exposure to each [DO] selected by the researcher^35^. Direct comparisons of CS and IF respirometry in corals have not been completed although evidence from fish suggest that critical phenotypes generated from the two approaches may not be directly comparable^71^.

### Experimental execution

Biotic, abiotic, and technical variation during experimental execution can influence the observed coral responses to deoxygenation within a given study. When unaccounted for, these methodological choices can prevent direct comparisons between studies but are rarely reported in published methods. While these variables of experimental execution are not frequently tested within deoxygenation experimental designs, evidence from experiments examining biotic and abiotic effects on coral oxygen and stress physiology (e.g., temperature^72^, circadian rhythm^36^, or feeding^73^) justify concerns that differences in experimental environments and sampling schemes can confound cross-study comparisons.

#### Experimental environment

During experimental execution, choices in temperature, salinity, light, and hydrodynamic flow can accumulate in effects on O_2_ availability and O_2_ consumption. For instance, water flow regulates gas exchange between water and the atmosphere, maintains mixing of oxygenated water masses, and impacts gas exchange between corals and the surrounding seawater via a coral’s DBL^44,45^. Given reduced flow will increase DBL thickness and thus decrease the diffusive O_2_ supply from the surrounding water to the coral tissue, low or unstable flow can increase the inherent disparity between the [DO] measured in the bulk seawater and that within the coral tissue^1,56,74^. Despite the role of flow, descriptions of hydrodynamic regimes in deoxygenation experiments are often subjective, making it difficult to account for flow effects in experimental outcomes.

Measured values of [DO] will also be dependent on the factors that influence the solubility of O_2_ in water, i.e., temperature, pressure, and salinity^75^. When unreported, variation in experimental temperature, salinity, or pressure will manifest in an inability to account for difference in measured [DO] and actual [DO] as well as difficulty converting between units of [DO] (e.g., % air saturation, hPa, µmol O_2_ L^−1^, mL O_2_ mL^−1^, mg O_2_ mL^−1^). The positive influence of temperature and light on coral O_2_ consumption^1,72^ and O_2_ production^72,76^ highlights how experimental O_2_ availability can also be affected by other biological processes, as experimental environments differ within and across deoxygenation studies.

Using nitrogen gas (N_2_) to expel O_2_ from a water mass for deoxygenation experimentation also results in carbon dioxide expulsion that raises the pH of experimental systems. Closed system respirometry which relies on respiration-based deoxygenation^24–26,69^ also traps waste products, e.g., CO_2_, resulting in potential changes to water chemistry. Though the magnitude of these changes appears to be minimal^22,35^, their impact in assays with corals have only begun to be directly evaluated. Practical approaches for generating deoxygenated water have been developed previously^62–64^ and offer useful blueprints to generate and control experimental [DO] and pH.

Experimental environments also inherently include the conditions corals experience prior to measurements of hypoxia phenotypes. Stress from preparing corals for respirometry (e.g., the movement from tanks to chambers) may influence coral respiration state and therefore downstream critical phenotypes. While acclimation steps have been employed to minimize these effects, there is little consensus on the appropriate duration with periods ranging from 1 h^24,25,35^ to overnight^69^. Heterotrophic feeding can impact coral respiration rates^73,77^ and support the coral holobiont stress response and recovery^78,79^. Similarly, the addition of particulate organic matter during feeding can elevate background microbial respiration, further reducing experimental O_2_ availability^80^. While blanks in respirometry are regularly used to correct respiration rates, the contribution of background microbial respiration rates and feeding regime have not been explicitly tested in coral deoxygenation research. Moreover, components of aquarium maintenance, e.g., water filtration or turnover rate, are often reported without sufficient detail to be assessed through comparisons of independent studies.

#### Sampling scheme

The regular, nightly O_2_ depletion within coral tissues^1,7,8^ and on reefs broadly^3^ suggest exposure to low O_2_, and therefore physiological responses to hypoxia, will be tied to a circadian clock. Symptom and tolerance-modulating phenotypes evaluated across a diel cycle show large differences throughout nighttime deoxygenation^19,36,37,81^. Critical phenotypes have been evaluated in the morning^69^, noon^35^, and night^25^, but the effect of time-of-day is difficult to evaluate as these studies used different species and analytical approaches. When phenotypes are measured relative to the onset of hypoxia stress will also be relevant for interpreting responses. For example, transcriptomic responses during recovery rather than at peak deoxygenation exposure helped explain variation in bleaching for two Acroporid species^20^. Finally, spatial variation in critical phenotypes across coral colonies^26^ suggests that sampling location may also contribute to variation in coral responses to deoxygenation.

### Reporting summary

Further information on research design is available in the Nature Portfolio Reporting Summary linked to this article.

## Toward methodological standardization

A future framework for methodological standardization in coral deoxygenation experimentation should provide ways to keep experimental factors controlled between studies so outcomes can be compared. However, we acknowledge that the current state of the field cannot support the development of best-practices for many of the experimental aspects discussed above. Therefore, we provide recommendations to progress toward this framework of methodological standardization that either (1) use data to support a recommended approach or (2) identify key research areas for prioritization.

### Focal phenotypes

#### Recommendation 1: Regulation-based analysis offers the most robust approach for measuring critical phenotypes in corals

The assumptions used in the derivation of P_crit_ prevent proper application in corals (e.g., Fig. 2). So although P_crit_ is the most common critical phenotype assessed in aerobic organisms^22^, the regulation-based analysis that generates P_cmax_, is most appropriate for application in corals because it can reproduce P_crit_ when HRCs follow a biphasic shape (Fig. 2C) while also more closely evaluating aerobic thresholds for non-traditional HRC shapes (Fig. 2D). The method described in Cobbs and Alexander^21^ can accommodate and describe both positive and negative oxyregulation, allowing for most metabolic responses to progressive deoxygenation to be assessed.

While robust to HRC shape, the regulation-based method relies on selecting a model to represent potentially noisy respirometry data. Careful model selection is therefore necessary to avoid underfitting and overfitting data (*sensu* Dilernia et al.^26^). Similarly, calculations of oxyregulation rely on comparisons to the null (oxyconformer) model^21^ and variation in intercepts of the null will lead to variation in P_cmax_ and T_pos_^23^. Therefore, consideration of these sources of bias will be necessary for those incorporating the regulation-based method. Regardless of analytical approach, open access to raw O_2_ consumption data from respirometry measurements will help alleviate analytical divergence by enabling reanalysis of results for comparison across studies.

#### Recommendation 2: groundtruthing symptom phenotypes with effect size and in situ performance to identify common currencies to quantify coral hypoxia responses

Identifying highly responsive symptom phenotypes that are indicative of coral fitness under deoxygenation should be prioritized as ongoing experiments explore the diversity of coral hypoxia responses. Effect size quantifies the magnitude of a relationship between variables, rather than the statistical significance relative to a null, and can therefore provide a tool for comparing responsiveness of coral traits across experimental designs. If consistently reported across studies, effect sizes can help (1) identify the experimental designs, deoxygenation intensities, and deoxygenation durations required to elucidate intra- and interspecific variation, (2) uncover the types of deoxygenation stress that trigger various aspects of the hypoxia response, and (3) nominate candidate phenotypes most useful to evaluate susceptibility, resilience, or adaptability to deoxygenation in coral. Records of in situ coral performance during deoxygenation events can help associate experimental hypoxia responses with real-world coral fitness to strengthen the case for symptom phenotypes as predictive metrics for coral hypoxia tolerance.

#### Recommendation 3: Tolerance-modulating phenotypes offer more insight when evaluated in conjunction with critical or symptom phenotypes

Evaluating tolerance-modulating phenotypes in conjunction with critical and symptom phenotypes allows (1) variation in critical or symptom traits to be better explained with tolerance-modulating traits, (2) cost or benefits to be assigned to tolerance-modulating phenotypes in relation to the coral’s hypoxia response, and (3) opportunities to identify causal relationships between tolerance-modulating phenotypes and fitness under deoxygenation. Such parallel assessment can test the hypothesized roles of, for example, coral morphotype^41^ and polyp behavior^69^, to clarify how, when, and where tolerance-modulating phenotypes are adaptive or maladaptive.

#### Recommendation 4: Refining hypoxia thresholds as time- or dose- dependent thresholds

Sublethal and lethal thresholds in medical, eco-toxicology, and the broader marine hypoxia fields offer standardized formats to account for time and intensity of stress exposure^60,82,83^. Specifically, ED_50_ (effective dose), LC_50_ (lethal concentration), or LT_50_ (lethal time) describe the dose or time required to reduce a common trait to 50% of its initial value^82^. Alva Garica et al.^9^ provides an example of this format by calculating SLT_50_ (median sublethal bleaching time at 1.25 mg O_2_ L^−1^) as a sublethal hypoxia threshold for *Galaxea fascicularis*. In this format, the duration and intensity of deoxygenation as well as the cutoff for a diagnostic change are explicitly incorporated into the thresholds. We encourage the evaluation of standardized hypoxia thresholds in a number of symptom phenotypes given that it is still unclear which, if any, hypoxia thresholds predict fitness under deoxygenation in situ.

### Deoxygenation exposures

#### Recommendation 5: Selecting experimental conditions based on aerobic thresholds and in situ conditions

Given the onset of hypoxia in corals is species-specific^60,65^, identical deoxygenation intensities applied across studies would create disparate levels of experienced hypoxia. Instead, basing selection of experimental deoxygenation intensities on hypoxia thresholds or in situ O_2_ dynamics^12,18,28,31^ would create a flexible framework to balance species-specific responses and cross-study comparisons. For example, selecting deoxygenation intensities as a percentage of aerobic respiration thresholds or average in situ nightly minimums could allow studies to supply similar theoretical stress levels that are dependent on the coral’s respiratory physiology or native habitat.

#### Recommendation 6: Quantifying low-O_2_ stress to integrate deoxygenation intensity, duration, variability, and environmental history

Using metrics that can quantify total low-O_2_ stress by integrating intensity, duration, and variability would enable cross-study comparison via a composite value that can be applied in situ or within experiments. A composite metric has been used to account for intensity and duration of low O_2_ on reefs, where Pezner et al.^13^ calculated the “severity” of in situ deoxygenation events as the difference of environmental [DO] relative to a predetermined [DO] value, multiplied by the duration of exposure. Similarly, Lucey et al.^31^ used experimentally defined coral hypoxia responses to calculate in situ “hypoxia pressure” by identifying the number and duration of low-O_2_ events (when [DO] dropped below evaluated species-specific bleaching thresholds, i.e., 1 mg O_2_ L^−1^). In that case, hypoxia pressure was used to characterize the expected bleaching for a coral under each in situ low-O_2_ event and cumulatively for all events over the study period.

The approaches implemented in Pezner et al.^13^ and Lucey et al.^31^ operate on evidence that longer or more intense deoxygenation is more stressful. However, it is still unclear if stress from duration and intensity are interchangeable, e.g., will doubling the intensity for half the time produce similar stress within a coral? The relationship between intensity and duration will need to be evaluated (*sensu* Lucey et al.^31^) before divergent experimental designs, such as those with stable vs. fluctuating deoxygenation exposures, can be compared with a composite metric of total low-O_2_ stress.

#### Recommendation 7: exploration of rates of deoxygenation in coral hypoxia responses

The rate of deoxygenation and/or reoxygenation during experimental O_2_ manipulation may be of particular concern as more experiments incorporate fluctuating deoxygenation treatments. Investigation of coral hypoxia responses under varying rates of deoxygenation may help the design of experimental exposures, while also offering a useful exploration of the mechanisms and sensitivity of coral responses. Prior to dedicated experiments on the rate of deoxygenation, careful reporting of ramping rates and/or the duration of closed system respirometry trials will allow for ramping rate to be accounted for in cross-study comparisons, while also enabling meta-analysis of ramp rate on coral hypoxia responses.

### Experimental execution

#### Recommendation 8: Documentation of experimental environment

The need to replicate source habitats, acclimation conditions, or experimental targets in independent studies precludes the prescription of standardized temperature, light, salinity, flow, water quality, and feeding regimes for deoxygenation experiments. Instead, careful reporting of these aspects will be necessary for meta-analysis, cross-study comparability, and repeatability. Measurements of many experimental conditions are already standard practice^84^ however, measuring water velocity near to the coral tissue surface as well as bulk water movement and tank turnover can be useful to contextualize boundary layer flow dynamics important for O_2_ transport^4,56^. Maintenance protocols that also include the source of experimental seawater, filtration level, and frequency/volume of water changes in recirculating systems, and feeding volume, compositions, and frequency will be vital for cross-study comparison (see Supplementary Table 2). Given the expected change to pH during deoxygenation via N_2_ gas addition and closed system respirometry, documentation and control of pH will be vital to minimize confounding effects of water chemistry.

#### Recommendation 9: Minimum standards for acclimatization of corals prior to experimentation

Impacts of moving, handling, and feeding have been mitigated with periods of acclimation. Pre-experiment procedures outlined in Grottoli et al.^84^, e.g. 7–12 days of acclimation post fragmentation, offer useful starting points for best practices, especially for multi-day experiments evaluating symptom or tolerance-modulating phenotypes. For assessments of critical phenotypes, acclimation may be less dependent on fragmentation—Dilernia et al.^26^ showed critical phenotypes did not vary with post-fragmentation time in *Acropora loripes* - but more so on the immediate stress of handling for respirometry. Therefore, acclimation for corals moved to incubation chambers will allow metabolic rates to return to baseline levels especially if (1) coral receive a supply of oxygenated water and (2) acclimation occurs in the dark to allow photosynthesis to ramp down prior to measuring VO_2_. The effect of feeding on critical phenotypes has not been tested in corals, however, supplemental feeding increases respiration in corals^72,73,76,77^ and starvation prior to respirometry is standard practice in other invertebrates and fish^22,85,86^.

#### Recommendation 10: timing, location, and replication of sampling

In situ O_2_ minima typically occur at night suggesting hypoxia phenotypes assessed overnight would best reflect the deoxygenation exposure to which reef building coral have likely adapted or acclimatized^1,2,87^. Methods reporting sampling time relative to the duration of deoxygenation exposure and the time of day will help account for variation in timing. Intracolonial variation due to spatial patterns in hypoxia phenotypes^26^ may be accounted for if a coral fragment’s spatial origin (e.g., interior vs exterior, top vs. side) and/or morphology is documented. For replication at the biological resolution of interest (e.g., colony, genotype, species, population), researchers will want to consider analysis-specific standard practices. For critical phenotypes in corals, six replicates were sufficient to detect a significant difference in P_cmax_ between interior and exterior branches of *Acropora* cf. *microphthalma* colonies^26^.

### Limitations of a standardized methodological framework in coral deoxygenation

The field of coral deoxygenation is far behind that of coral heat stress making the framework outlined in Grottoli et al.^84^ aspirational rather than immediately achievable. The standardized framework proposed in Grottoli et al.^84^ is built on decades of experiments that generated knowledge such as (i) the reliable phenotypes to describe bleaching, (ii) quantification of the influence of ramping rate, or (iii) identification of Maximum Monthly Mean to describe in situ thermal environments. Therefore, the development of a standardized methodology in coral deoxygenation experiments must first be preceded by fundamental knowledge of the key biological and environmental components outlined above.

Even when supported by empirical evidence, the application of standardized approaches may not be practical or appropriate to address all questions in coral deoxygenation research, and a framework to compare all coral deoxygenation studies is not a reasonable expectation. For instance, studies exploring hypoxia responses under future ocean O_2_ conditions (amplified diel fluctuations in O_2_) vs. those under persistent anoxia typical of short-term low-O_2_ events address two ecologically relevant questions that must be tested with different experimental designs. However, if these two experiments overlapped in some methodological components (i.e., the focal phenotypes), their comparison could still yield insights into the triggers of hypoxia, quantification of deoxygenation stress, or how current stress events can predict future winners. Therefore, rather than homogenizing all deoxygenation studies, a constructive framework would emphasize creating opportunities to integrate otherwise independent studies through common phenotypes, O_2_ exposures, or experimental execution.

### Consequences of waiting to develop a standardized framework

Tangible consequences of the methodological divergence in coral deoxygenation research may seem rare given most cross-study comparisons are limited to discussions among studies^18,25,35^ and brief data summaries^9,65^. However, coral deoxygenation research is on a path similar to many other fields: (1) rapid knowledge generation accompanied by methodological divergence, (2) attempts at cross-study comparison, meta-analysis, and application that reveal gaps in methodologies, and (3) efforts to standardized methodology to produce comparable research. Examples in topics as broad as oceanographic observations^88^ or psychological tests^89^ and as narrow as reporting experimental data using microarray technology^90^ or decision-making experiments in mice^91^ highlight the seemingly ubiquitous eventuality of calls for methodological standardization following years of divergent research approaches. Therefore, the lack of confounded cross-study comparisons in coral deoxygenation is a result of its nascent stage rather than a sign that methodological divergence is a non-issue.

Standardization within coral deoxygenation methodology stands to extend its impact into other systems that experience similar dynamics and constraints on O_2_ availability. Cobbs and Alexander^21^ document the diversity of organisms that do not follow traditional oxyregulation profiles suggesting improvements in defining aerobic thresholds beyond P_crit_ will be applicable beyond coral (Recommendation 1). Efforts to characterize low-O_2_ stress as functions of intensity, duration, variability, and environmental history (Recommendation 6) will help quantify experimental and in situ deoxygenation for reef organisms and ecosystems susceptible to low-O_2_ stress.

With the growing threat of deoxygenation to corals and reef ecosystems^13,65^, each year that researchers, policy makers, and reef managers cannot incorporate knowledge of O_2_ and its consequences for reefs represents a critical loss of opportunity in the coral crisis. The methodological differences outlined here reveal the existing barriers to cross-study comparison in coral deoxygenation experimentation and confirm that the field’s trajectory mirrors that of many others^88–91^. Rather than waiting for the confounded meta-analyses, contradictory research outcomes, or slowed restoration that has precipitated calls for methodological standardization in other coral fields (reviewed in refs. ^15,92,93^), taking steps now to identify best practices and critical knowledge gaps in coral deoxygenation experimentation can help shorten the time to collective knowledge generation.

### Conclusion and next steps

**Fig. 5:**
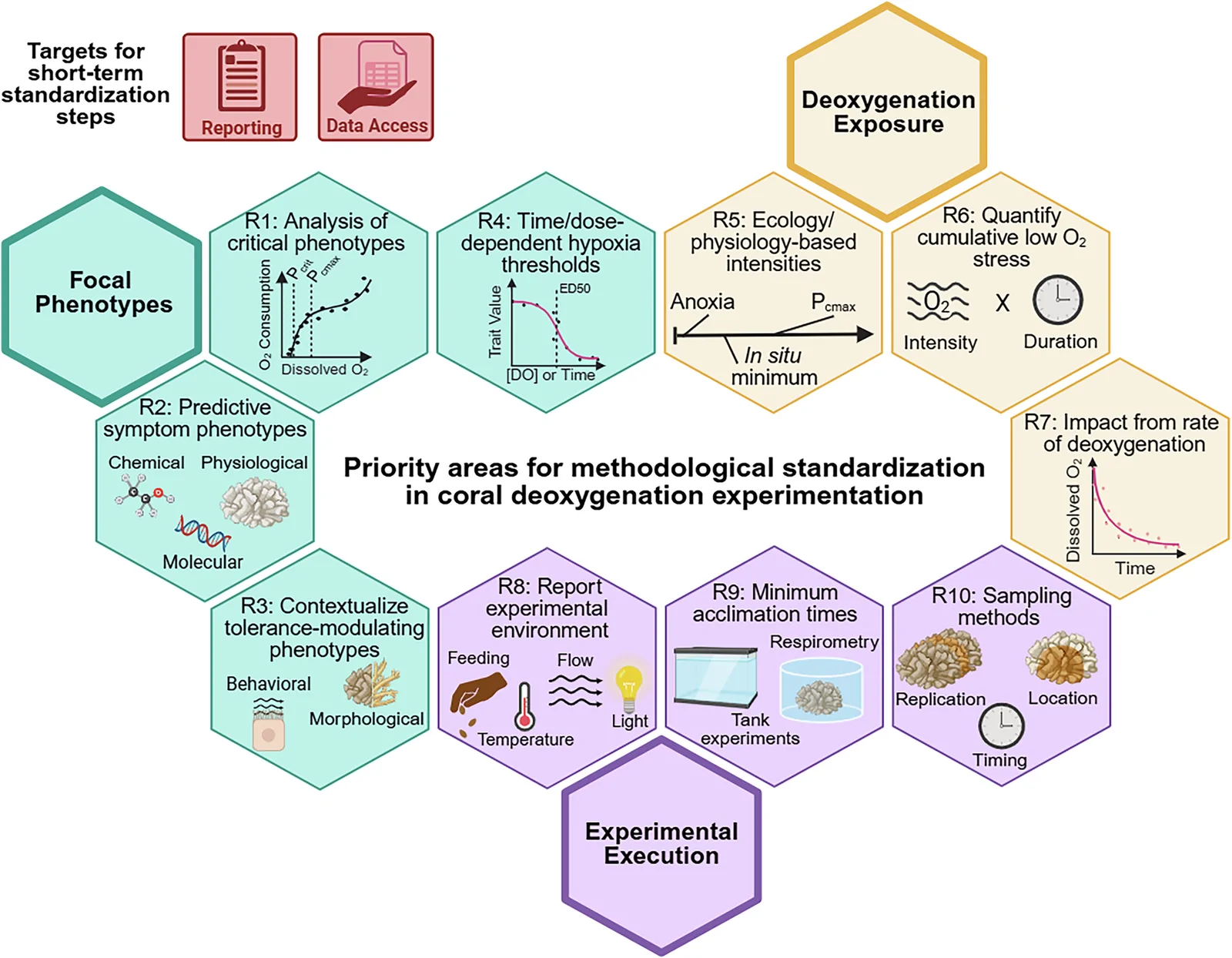
Recommendations to move toward standardization in coral deoxygenation experimentation. Proposed recommendations that will support establishment of a standardized framework for the field of coral deoxygenation experimentation to enable collective knowledge generation. Robust methods reporting and data sharing are immediate actions researchers can take to support standardization efforts. Created in BioRender. Million, W. (2026) https://BioRender.com/ucownbv.

Methodological differences in focal phenotypes, deoxygenation exposures, and experimental execution hinder our ability to identify broad trends, detect susceptible species or populations, and inform strategies to address the global coral crisis. Although in its early stages, prioritizing key topical areas in coral deoxygenation research will not only improve fundamental knowledge but will kickstart the formation of a framework that improves the translation of independent studies into collective knowledge. As key methodological knowledge gaps are being filled, transparency of methods and availability of data can allow for accounting for technical variation and/or reanalysis of incongruent phenotypes (e.g., Fig. 5). Table S1 offers a checklist of considerations for designing and reporting new deoxygenation experiments with corals which may also serve as a reference during peer-review. Collectively, the short- and long-term steps outlined above will allow the rapid mobilization toward standardized coral deoxygenation methodology that will be required to provide solutions to the urgent threat of deoxygenation on coral reefs.

## Supplementary information

Transparent Peer Review file

Supplementary Information

Description of Additional Supplementary files

Supplementary Data 1

Supplementary Data 2

Supplementary Data 3

Supplementary Data 4

Supplementary Data 5

Supplementary Data 6

Reporting summary

## Data Availability

No new data were created in this study but all scripts and reused published data are available as Supplementary Data 1–6 and at https://github.com/wyattmillion/CoralDeoxygenationPerspective

## Statement of Inclusion

Our study was a global review and was based on published work rather than primary data. As such, there was no local data collection. However, the geographical distribution of the authorship team broadly represents the major regions of interest in the review and effort was made to include relevant peer-reviewed studies without bias on region ensuring the appropriate representation of the available current state of knowledge in coral deoxygenation experimentation.
