# Supplementary material for: Standardizing deoxygenation studies in coral biology
Source: Commun Biol. 2026 Oct 19;9:1367. doi: 10.1038/s42003-026-11025-x (PMC13680498; doi:10.1038/s42003-026-11025-x)
Supplement: Supplementary file 1 [file 42003_2026_11025_MOESM10_ESM.pdf]

Corresponding author(s): Wyatt C MIllion

Last updated by author(s): Jun 12, 2026

Reporting Summary

Nature Portfolio wishes to improve the reproducibility of the work that we publish. This form provides structure for consistency and transparency in reporting. For further information on Nature Portfolio policies, see our Editorial Policies and the Editorial Policy Checklist.

Statistics

For all statistical analyses, confirm that the following items are present in the figure legend, table legend, main text, or Methods section.

n/a	Confirmed	
☐	☒	The exact sample size (*n*) for each experimental group/condition, given as a discrete number and unit of measurement
☒	☐	A statement on whether measurements were taken from distinct samples or whether the same sample was measured repeatedly
☒	☐	The statistical test(s) used AND whether they are one- or two-sided<br>*Only common tests should be described solely by name; describe more complex techniques in the Methods section.*
☒	☐	A description of all covariates tested
☒	☐	A description of any assumptions or corrections, such as tests of normality and adjustment for multiple comparisons
☒	☐	A full description of the statistical parameters including central tendency (e.g. means) or other basic estimates (e.g. regression coefficient) AND variation (e.g. standard deviation) or associated estimates of uncertainty (e.g. confidence intervals)
☒	☐	For null hypothesis testing, the test statistic (e.g. F, t, r) with confidence intervals, effect sizes, degrees of freedom and P value noted<br>*Give P values as exact values whenever suitable.*
☒	☐	For Bayesian analysis, information on the choice of priors and Markov chain Monte Carlo settings
☒	☐	For hierarchical and complex designs, identification of the appropriate level for tests and full reporting of outcomes
☒	☐	Estimates of effect sizes (e.g. Cohen's d, Pearson's r), indicating how they were calculated

Our web collection on statistics for biologists contains articles on many of the points above.

Software and code

Policy information about availability of computer code

Data collection	no new data was collected in this study
Data analysis	R Core Environment, R packages: ggplot2, tidyverse, drc, respirometry, svglite

For manuscripts utilizing custom algorithms or software that are central to the research but not yet described in published literature, software must be made available to editors and reviewers. We strongly encourage code deposition in a community repository (e.g. GitHub). See the Nature Portfolio guidelines for submitting code & software for further information.

Data

Policy information about availability of data

All manuscripts must include a data availability statement. This statement should provide the following information, where applicable:

- Accession codes, unique identifiers, or web links for publicly available datasets
- A description of any restrictions on data availability
- For clinical datasets or third party data, please ensure that the statement adheres to our policy

No new data were created in this study but all scripts and reused published 721 data are available at https://github.com/wyattmillion/CoralDeoxygenationPerspective

Research involving human participants, their data, or biological material

Policy information about studies with human participants or human data. See also policy information about sex, gender (identity/presentation), and sexual orientation and race, ethnicity and racism.

Reporting on sex and gender	n/a
Reporting on race, ethnicity, or other socially relevant groupings	n/a
Population characteristics	n/a
Recruitment	n/a
Ethics oversight	n/a

Note that full information on the approval of the study protocol must also be provided in the manuscript.

Field-specific reporting

Please select the one below that is the best fit for your research. If you are not sure, read the appropriate sections before making your selection.

☐ Life sciences ☐ Behavioural & social sciences ☒ Ecological, evolutionary & environmental sciences

For a reference copy of the document with all sections, see nature.com/documents/nr-reporting-summary-flat.pdf

Ecological, evolutionary & environmental sciences study design

All studies must disclose on these points even when the disclosure is negative.

Study description	The current manuscript is a perspective piece that includes one figure (Figure 2) that includes previously published data from Hughes et al. 2022 Widespread oxyregulation in tropical corals under hypoxia. The reanalysis of a portion of this data - a single replicate from two species of coral evaluated in the study - applies two forms of analysis of aerobic thresholds to compare how the outputs of the analyses differ and create bias. The manuscript uses this reanalysis to support an argument that the ways in which we analyze aerobic thresholds in coral deoxygenation biology requires standardization. The number of publications in coral deoxygenation research over time was generated from a Web of Science search using topic codes “coral” AND “deoxygenation” OR “hypoxia” OR ”hypoxic” produced 365 publications. This list was manually filtered to remove duplicates and articles focusing on hypoxia in non-scleractinian organisms (e.g., studies in “coral-reef fish”). Articles were counted if they included in situ or ex situ investigation of the coral holobiont, coral biotic interactions (e.g., coral-macroalgae O2 competition), or coral reef ecosystems.
Research sample	The reanalyzed data include one replicate of Porites lutea and one replicate of Hydnophora rigida under standard conditions with no treatment effect or any other experimental condition.
Sampling strategy	The data were sampled following the methods outlined in Hughes et al. 2022 Widespread oxyregulation in tropical corals under hypoxia. When accessed, the dataset was in a format of dissolved oxygen concentration over time - representing the incubation of a coral within a respirometry chamber as it consumed oxygen over a period of time. From the DO vs. time data set, the current study calculated oxygen consumption rate then applied the two aerobic threshold analyses (for Pcrit and Pcmax) on the VO2 vs. DO relationship. Pcrit was analyzed within the respirometry package. Pcmax was assessed following Cobbs & Alexander 2018 Assessment of oxygen consumption in response to progressive hypoxia. The R code used to complete the Pcmax analysis is included in the github repository linked in the Data Availability statement.
Data collection	Data collection ifor the data used for reanalysis is described in Hughes et al. 2022 Widespread oxyregulation in tropical corals under hypoxia. The number of publications in coral deoxygenation research over time was generated from a Web of Science search using topic codes “coral” AND “deoxygenation” OR “hypoxia” OR ”hypoxic” produced 365 publications. This list was manually filtered to remove duplicates and articles focusing on hypoxia in non-scleractinian organisms (e.g., studies in “coral-reef fish”). Articles were counted if they included in situ or ex situ investigation of the coral holobiont, coral biotic interactions (e.g., coral-macroalgae O2 competition), or coral reef ecosystems.
Timing and spatial scale	Data collection for the data used for reanalysis is described in Hughes et al. 2022 Widespread oxyregulation in tropical corals under hypoxia.
Data exclusions	No data was excluded from the portion reanalyzed from Hughes et al. 2022 Widespread oxyregulation in tropical corals under hypoxia. The portion of the data from Hughes et al. 2022 was selected to represent diverging HRC shapes that exist in coral.
Reproducibility	Reproducibility of data collection for the data used for reanalysis is described in Hughes et al. 2022 Widespread oxyregulation in tropical corals under hypoxia.

Randomization	Randomization of data collection for the data used for reanalysis is described in Hughes et al. 2022 Widespread oxyregulation in tropical corals under hypoxia.
Blinding	Any blinding of data collection for the data used for reanalysis is described in Hughes et al. 2022 Widespread oxyregulation in tropical corals under hypoxia.

Did the study involve field work? ☐ Yes ☒ No

Reporting for specific materials, systems and methods

We require information from authors about some types of materials, experimental systems and methods used in many studies. Here, indicate whether each material, system or method listed is relevant to your study. If you are not sure if a list item applies to your research, read the appropriate section before selecting a response.

Materials & experimental systems

n/a	Involved in the study
☒	☐ Antibodies
☒	☐ Eukaryotic cell lines
☒	☐ Palaeontology and archaeology
☒	☐ Animals and other organisms
☒	☐ Clinical data
☒	☐ Dual use research of concern
☒	☐ Plants

Methods

n/a	Involved in the study
☒	☐ ChIP-seq
☒	☐ Flow cytometry
☒	☐ MRI-based neuroimaging

Plants

Seed stocks	*Report on the source of all seed stocks or other plant material used. If applicable, state the seed stock centre and catalogue number. If plant specimens were collected from the field, describe the collection location, date and sampling procedures.*
Novel plant genotypes	*Describe the methods by which all novel plant genotypes were produced. This includes those generated by transgenic approaches, gene editing, chemical/radiation-based mutagenesis and hybridization. For transgenic lines, describe the transformation method, the number of independent lines analyzed and the generation upon which experiments were performed. For gene-edited lines, describe the editor used, the endogenous sequence targeted for editing, the targeting guide RNA sequence (if applicable) and how the editor was applied.*
Authentication	*Describe any authentication procedures for each seed stock used or novel genotype generated. Describe any experiments used to assess the effect of a mutation and, where applicable, how potential secondary effects (e.g. second site T-DNA insertions, mosiacism, off-target gene editing) were examined.*
