# Supplementary material for: Standardizing deoxygenation studies in coral biology
Source: Commun Biol. 2026 Oct 19;9:1367. doi: 10.1038/s42003-026-11025-x (PMC13680498; doi:10.1038/s42003-026-11025-x)
Supplement: Supplementary file 2 [file 42003_2026_11025_MOESM1_ESM.pdf]

nature portfolio

Peer Review File

Standardizing deoxygenation studies in coral biology

Corresponding Author: Dr Wyatt Million

This file contains all reviewer reports in order by version, followed by all author rebuttals in order by version.

Version 0:

Reviewer comments:

Reviewer #1

(Remarks to the Author)
The manuscript presents an extensive and timely perspective on methodological divergence in coral deoxygenation research. It synthesizes evidence from recent studies and provides recommendations aimed at standardizing focal phenotypes, deoxygenation exposures, and experimental execution in coral biology. The authors argue that methodological inconsistency currently prevents meaningful cross-study comparison, thereby slowing progress in understanding coral responses to deoxygenation, a rapidly intensifying environmental threat.
Overall, the manuscript is well-written, thorough, and well-positioned to influence the field. It fills a gap by highlighting the lack of standardized approaches in coral deoxygenation research and proposing a structured path toward standardization. This manuscript is likely to have a lasting impact. The paper should be published after addressing some minor clarifications and providing slightly more justification for key recommendations. Please see comments below.

1. Validation of regulation-based methods (Pcmax) in corals
While the authors convincingly show that Pcrit is problematic for corals, the claim that Pcmax is “most appropriate” would be strengthened by:
a. More explicit benchmarking across taxa or analytic simulations (e.g, Ultsch, G.R. & Regan, M.D. (2019). The utility and determination of Pcrit in fishes. Journal of Experimental Biology, 222: jeb210633)
b. Discussion of scenarios where Pcmax may also be biased (e.g., under metabolic depression).(Guppy, M. & Withers, P. (1999). Metabolic depression in animals: physiological perspectives and biochemical generalizations. Biological Reviews, 74, 1–40.)

2. Evidence that symptom phenotypes (e.g. bleaching metrics, photochemical efficiency) correlate with long-term fitness.The authors acknowledge this limitation but could further reinforce:
a. Which symptom metrics appear most consistently responsive,
b. How effect sizes could be used to build consensus.

3. Interpretation of tolerance-modulating phenotypes
This section is conceptually rich but would benefit from:
a. Clarifying when these phenotypes are causal vs. correlative (Rezende, E.L. et al. (2014). Thermal tolerance landscapes and the evolution of thermal limits. Proceedings of the Royal Society B, 281, 20141076.
Sunday, J.M. et al. (2011). Global analysis of thermal tolerance and latitude. Proceedings of the Royal Society B, 278, 1823–1830.),
b. Specific examples linking tolerance-modulating traits to measurable fitness under deoxygenation.

4. Terminology Section (Table 1). This is excellent, but several definitions refer to “present study.” It may be helpful to explicitly state when definitions are newly proposed vs. synthesized. Consider clarifying “deoxygenation intensity” using both concentration and % air saturation for accessibility.

5. Figure 2 (3D conceptual map) Very useful, but the message could be stronger with a brief text box summarizing common pitfalls (e.g., mismatched phenotypes across studies).
For example, Figure 2 message would be strengthened by explicitly summarizing the methodological pitfalls that arise when studies differ along these dimensions. As it stands, readers must infer why variation on these axes undermines comparability. A brief text box could therefore help articulate the core problem: that mismatches in what is measured (e.g.,

photophysiology vs. gene expression), how hypoxia is delivered (e.g., different O_2 intensities, durations, or fluctuating vs. constant exposures), and how experiments are implemented (e.g., flow regimes, light cycles, or acclimation times) create incompatible datasets that cannot be meaningfully integrated. Such divergence often stacks across multiple axes simultaneously, making it impossible to determine whether observed differences among studies reflect true biological variation or artifacts of inconsistent methods.

6. Section on variability (fluctuating vs. constant O_2). This section could benefit from a stronger link to in situ diel patterns and field data variability ranges. For example see Fusi et al (2025) https://www.sciencedirect.com/science/article/pii/S0169534725001636?via=ihub. Particularly the manuscript could benefit further from introducing flows and fluxes as important components of species O2 availability and interactions. See for example the Figure 1 of Fusi et al 2025, but also Wilko Verberk works (e.g., https://esajournals.onlinelibrary.wiley.com/doi/abs/10.1890/10-2369.1). The authors emphasize flow and DBL dynamics, but a brief practical suggestion on how to measure or characterize flow (e.g., average bulk velocity or turbulence measurements) would support reproducibility and also how flow links to O2 concentrations to generate supply for marine species that liv around corals.

end of review

Reviewer #2

(Remarks to the Author)
This is a nice review documenting an early call-to-action to standardize coral deoxygenation experiments to accelerate collective knowledge of this emerging coral reef stressor. It is timely, well-researched, and represents an important contribution to the broader coral deoxygenation literature. I have just a few comments to consider in their revisions.

While this review focuses on coral biology, many of these same considerations would be equally valid for other coral reef organisms, which could have interactive effects on coral biology under deoxygenation. The authors may choose to explicitly mention this somewhere in the review.

Table 1: The authors appear to separate deoxygenation intensity as [DO] whereas hypoxia thresholds could be defined based on intensity and/or duration. I see the logic here, but wouldn’t deoxygenation duration and/or severity (i.e., intensity*duration) also be a key variable(s) to differentiate separate here? I see this is later discussed in Recommendation 6, but I think the authors should consider whether this should be explicitly defined in Table 1.

Experimental Execution and Recommendations: I feel that these sections are missing an explicit discussion for how low DO treatments are generated and recommendations for standardizing low DO conditions in experimental aquaria. For example, some studies simply removing O2 with N2 gas without a discussion for how CO2 gas is controlled to avoid confounding deoxygenation effects with those of CO2-chemistry? Do we care about isolating O2 conditions from CO2 conditions if in reality these are highly correlated in situ? I see this is listed in Table S1 but I think this should be discussed in greater detail in the main MS.

Line 681: missing parentheses closure

Version 1:

Reviewer comments:

Reviewer #1

(Remarks to the Author)
I have carefully reviewed the revised version of the manuscript and the authors’ responses to the reviewers’ comments. The authors have adequately addressed all the issues raised in the first round, and the manuscript has been substantially improved in terms of clarity, methodology, and presentation. In my opinion, the manuscript now meets the standards required for publication, and I recommend its acceptance in its current form.

Reviewer #2

(Remarks to the Author)
The authors have addressed all of my previous concerns. I found just a few grammar suggestions to incorporate into the final version:
Line 23: “reef building coral” to “reef building corals”
Line 433: “conditions coral experiences” to “the conditions corals experience”
Line 502 and 513: add comma after “response”
Line 584: “turn over” to “turnover”

Open Access This Peer Review File is licensed under a Creative Commons Attribution 4.0 International License, which permits use, sharing, adaptation, distribution and reproduction in any medium or format, as long as you give appropriate credit to the original author(s) and the source, provide a link to the Creative Commons license, and indicate if changes were made.
In cases where reviewers are anonymous, credit should be given to 'Anonymous Referee' and the source.
The images or other third party material in this Peer Review File are included in the article's Creative Commons license, unless indicated otherwise in a credit line to the material. If material is not included in the article's Creative Commons license and your intended use is not permitted by statutory regulation or exceeds the permitted use, you will need to obtain permission directly from the copyright holder.
To view a copy of this license, visit https://creativecommons.org/licenses/by/4.0/

Thank you to both reviewers and the editor for the thoughtful suggestions. We were able to address the majority of the comments with additional clarifying text where needed in the manuscript. Please see the response to review below (reviewer comments in blue and author responses in black). Line numbers mentioned below are taken from the full track changes document that is included with the resubmission. Additionally, to address the note from the editor, the Figure 1 as been move to supplementary material and a brief summary of the trend was mention in the text as a replacement for the figure.

Reviewer #1 (Remarks to the Author):

The manuscript presents an extensive and timely perspective on methodological divergence in coral deoxygenation research. It synthesizes evidence from recent studies and provides recommendations aimed at standardizing focal phenotypes, deoxygenation exposures, and experimental execution in coral biology. The authors argue that methodological inconsistency currently prevents meaningful cross-study comparison, thereby slowing progress in understanding coral responses to deoxygenation, a rapidly intensifying environmental threat.
Overall, the manuscript is well-written, thorough, and well-positioned to influence the field. It fills a gap by highlighting the lack of standardized approaches in coral deoxygenation research and proposing a structured path toward standardization.
This manuscript is likely to have a lasting impact. The paper should be published after addressing some minor clarifications and providing slightly more justification for key recommendations. Please see comments below.

1. Validation of regulation-based methods (Pcmax) in corals
While the authors convincingly show that Pcrit is problematic for corals, the claim that Pcmax is "most appropriate" would be strengthened by:
a. More explicit benchmarking across taxa or analytic simulations (e.g, Ultsch, G.R. & Regan, M.D. (2019). The utility and determination of Pcrit in fishes. Journal of Experimental Biology, 222: jeb210633)

We agree that benchmarking is important for the argument for using Pcmax. The authors of the method in Cobbs & Alexander 2018 (https://doi.org/10.1371/journal.pone.0208836) explore this by assessing the goodness of fit across hypoxia response curves from respirometry of 68 animals from14 species from multiple phyla for the traditional biphasic (Pcrit) models and 11 other descriptive models. Their results show that few animals fit the biphasic model best, suggesting if Pcrit was used to define an aerobic threshold, the resulting threshold would not be representative of the organisms true oxyregulation strategy. The regulation based (i.e., Pcmax) framework includes the biphasic model as an option, but selects a model based on fit rather than traditional assumptions suggesting it is more robust of an approach.

Cobbs and Alexander stop short of comparing Pcmax to Pcrit directly as we have done here, however, extending the analysis seen in Figure 3 (now figure 2) is beyond the scope of the article and pushes the journal's policies for presenting new data within perspectives. However, this benchmarking via reanalysis is a important step in validating aerobic thresholds and we hope will be a product of the ideas this perspective highlights.

We make the outcomes from Cobbs & Alexander 2018 more explicit within the text (Line 200-203) and include this preliminary benchmarking across taxa from this study when discussing the broader impacts of standardization in the coral field (Line 698-701).

b. Discussion of scenarios where Pcmax may also be biased (e.g., under metabolic depression).(Guppy, M. & Withers, P. (1999). Metabolic depression in animals: physiological perspectives and biochemical generalizations. Biological Reviews, 74, 1–40.)

This is a more appropriate avenue to include than the meta-analysis mentioned above and we agree this discussion is necessary to include in the manuscript. Pcmax relies on fitting a model to the hypoxia response data creating opportunities for bias via over simplification or over fitting. These consequences of using Pcmax were evaluated in Dilernia et al. 2024 (https://doi.org/10.1002/ece3.11100) but were limited to a few coral species. Cobbs and Alexander 2018 (https://doi.org/10.1371/journal.pone.0208836) provide a robust evaluation of the regulation-based analysis on HRC shape. However, as discussed in Regan et al. 2019 (https://doi.org/10.1242/jeb.200253), there is potential for biased based on where the initial VO2 (MO2std in their terms) is placed to anchor the oxyconformity line – as regulation value is based on the divergence between he HRC and oxyconformity line, the position of this line becomes critical to the analysis.

We changed the text in recommendation 1 (Line 508-518), to reflect these considerations with a greater explanation of the regulation-based method and how model selection, which includes issues of over and under fitting as well as anchoring the null (i.e., the intercept), can influence outcomes.

Similarly, a more explicit mention of metabolic depression when discussing the flexibility of the regulation-based method was included (Line 193-194) to make it clear Figure 2 already shows this common type of oxyregulation strategy.

2. Evidence that symptom phenotypes (e.g. bleaching metrics, photochemical efficiency) correlate with long-term fitness. The authors acknowledge this limitation but could further reinforce:

a. Which symptom metrics appear most consistently responsive,

This is a great question but in the absence of comparable experimental designs, assessments of responsiveness are difficult. For example, bleaching metrics like Fv/Fm and color loss are commonly seen in lethal experiments but changes to photosynthetic health are often absent in long-term sublethal deoxygenation. Moreover, assessment of

responsiveness at this point will likely be biased by sampling frequency in that phenotypes like gene expression will likely be consistently responsive (given they underlie higher-order trait variation) but have only been explored in two of the study include at the time of submission, this is in addition to the high variation in exposure intensity and duration between studies. This is another argument for effect sizes which are theoretically less biased on number of observations. However, short of a meta-analysis, we do not feel comfortable providing a cursory assessment of the most responsive phenotypes.

We did include an addition statement (Line 232-235) that provides evidence for reductions in bleaching related traits to be related to long-term fitness in coral under other forms of stress will also providing some examples of how changes in these traits under experimental deoxygenation also corroborate in situ performance.

b. How effect sizes could be used to build consensus.
To better address this point while still keeping the recommendations concise, we revised Recommendation 2 (Line 528-535) to provide a logical step from effect sizes to consensus building by explaining how effect sizes can be used to compare divergent experimental designs. Then, once these designs have a way to be more appropriately compared, their comparison can reveal important pieces of information to build a standardized framework – i.e., identifying experimental designs most capable of uncovering variation, pinpointing the types of deoxygenation stress (short vs. long term, sublethal vs lethal) that has stronger effects on different parts of the hypoxia response system, and identifying phenotypes most consistently responsive as mentioned above. We feel this revision of Recommendation 2 keeps it in line with the scope of the perspective to speak to methodological recommendations without the prescribing choices for actual experimental designs.

3. Interpretation of tolerance-modulating phenotypes
This section is conceptually rich but would benefit from:
a. Clarifying when these phenotypes are causal vs. correlative (Rezende, E.L. et al. (2014). Thermal tolerance landscapes and the evolution of thermal limits. Proceedings of the Royal Society B, 281, 20141076.
Sunday, J.M. et al. (2011). Global analysis of thermal tolerance and latitude. Proceedings of the Royal Society B, 278, 1823–1830.),
b. Specific examples linking tolerance-modulating traits to measurable fitness under deoxygenation.

This is an important point that we may not be in a position to full address within this manuscript – in part because examples of tolerance-modulating traits being measured in association with traditional fitness metrics in coral are somewhat rare. However, to incorporate this discussion, we added explicit discussion of stronger examples of

tolerance-modulating traits improving fitness under deoxygenation while also including an example of when a potential tolerance-modulating trait may likely be correlative (Line 290-308). This makes an even stronger justification for Recommendation 3 which we amended to include a mention of causal evaluations (Line 545-546) so we are appreciate this point being brought up.

4. Terminology Section (Table 1). This is excellent, but several definitions refer to "present study." It may be helpful to explicitly state when definitions are newly proposed vs. synthesized. Consider clarifying "deoxygenation intensity" using both concentration and % air saturation for accessibility.

In the original manuscript, definitions in table 1 that were taken from source material include citations at the end of the definition. For those proposed in the current study we included "(Present study)". However, definitions like deoxygenation intensity highlights represent a synthesis of multiple uses from the literature so we have included reference to those examples as needed in Table 1). Table 1 now also includes greater detail regarding units for deoxygenation intensity.

5. Figure 2 (3D conceptual map) Very useful, but the message could be stronger with a brief text box summarizing common pitfalls (e.g., mismatched phenotypes across studies).
For example, Figure 2 message would be strengthened by explicitly summarizing the methodological pitfalls that arise when studies differ along these dimensions. As it stands, readers must infer why variation on these axes undermines comparability. A brief text box could therefore help articulate the core problem: that mismatches in what is measured (e.g., photophysiology vs. gene expression), how hypoxia is delivered (e.g., different O_2 intensities, durations, or fluctuating vs. constant exposures), and how experiments are implemented (e.g., flow regimes, light cycles, or acclimation times) create incompatible datasets that cannot be meaningfully integrated. Such divergence often stacks across multiple axes simultaneously, making it impossible to determine whether observed differences among studies reflect true biological variation or artifacts of inconsistent methods.
A more explicit statement of consequences of would be useful but as we began creating a box, we did not find it warranted its own display item. This is because the open paragraph of the "Methodological divergence in coral deoxygenation experimentation" states much of the same thing:

"A growing body of evidence shows that focal phenotypes (e.g., aerobic respiration, behavior, mortality), experimental design (e.g., deoxygenation intensity or timing), and experimentation execution (sampling scheme or abiotic conditions) can each affect experimental outcomes in coral deoxygenation research. Currently, however, most studies simultaneously vary the measured focal phenotype, deoxygenation exposures, and

experimental execution in addition to the biological components (e.g. species, population, life stage, or symbiont community) making it difficult to unequivocally attribute observed outcomes to biological variance, technical variation, or both".

Therefore, we added an additional statement to the caption in Figure 2 (now Fig. 1) to explicitly restate this consequence of mismatched experiments. This occurs immediately before the discussion of comparisons seen in the figure which (to a degree) provide solutions to this core problem.

6. Section on variability (fluctuating vs. constant O_2). This section could benefit from a stronger link to in situ diel patterns and field data variability ranges. For example see Fusi et al (2025) https://www.sciencedirect.com/science/article/pii/S0169534725001636?via=ihub. Particularly the manuscript could benefit further from introducing flows and fluxes as important components of species O2 availability and interactions. See for example the Figure 1 of Fusi et al 2025, but also Wilko Verberk works (e.g., https://esajournals.onlinelibrary.wiley.com/doi/abs/10.1890/10-2369.1). The authors emphasize flow and DBL dynamics, but a brief practical suggestion on how to measure or characterize flow (e.g., average bulk velocity or turbulence measurements) would support reproducibility and also how flow links to O2 concentrations to generate supply for marine species that liv around corals.

We added a portion the start of the variability section (Line 377-385) to provide context for the experimental durations and variabilities reported later in the section. However, these concepts are better explored in other articles, like those suggested here, so to keep the scope of the manuscript focused on methodology, we believe this revision is appropriate.

We also extended Recommendation 8 to include a brief suggestion for measuring water velocity with the caveat of including tissue level flow in addition to bulk water velocity (Line 614-617).

end of review

Reviewer #2 (Remarks to the Author):

This is a nice review documenting an early call-to-action to standardize coral deoxygenation experiments to accelerate collective knowledge of this emerging coral

reef stressor. It is timely, well-researched, and represents an important contribution to the broader coral deoxygenation literature. I have just a few comments to consider in their revisions.

While this review focuses on coral biology, many of these same considerations would be equally valid for other coral reef organisms, which could have interactive effects on coral biology under deoxygenation. The authors may choose to explicitly mention this somewhere in the review.

We added a short paragraph for the broader implications of standardization for experimentation in reef organisms within the "Consequences of waiting .." section (Line 697-704). This is a useful contribution as it strengthens the urgency argument within the perspective. Thank you!

Table 1: The authors appear to separate deoxygenation intensity as [DO] whereas hypoxia thresholds could be defined based on intensity and/or duration. I see the logic here, but wouldn't deoxygenation duration and/or severity (i.e., intensity*duration) also be a key variable(s) to differentiate separate here? I see this is later discussed in Recommendation 6, but I think the authors should consider whether this should be explicitly defined in Table 1.

This is a very good point! Thank you for the recommendation as this is a key distinction that we didn't think to add but makes perfect sense to do so. Please see the added definitions in Table 1. However, we do not go as far to define the metric for severity beyond saying it will be a function of duration and intensity and providing an example of its use in the literature. We do not believe there is sufficient work to identify the relative contribution of the two components within that interaction.

Experimental Execution and Recommendations: I feel that these sections are missing an explicit discussion for how low DO treatments are generated and recommendations for standardizing low DO conditions in experimental aquaria. For example, some studies simply removing O_2 with N_2 gas without a discussion for how CO_2 gas is controlled to avoid confounding deoxygenation effects with those of CO_2-chemistry? Do we care about isolating O_2 conditions from CO_2 conditions if in reality these are highly correlated in situ? I see this is listed in Table S1 but I think this should be discussed in greater detail in the main MS.

Funnily enough, previous versions of the manuscript included sections devoted to this but were asked removed to keep focus on coral deoxygenation rather than principals of deoxygenation experimentation that are present and explored in other fields. However, there is capacity to discuss the common pitfalls of generating deoxygenated conditions. To address , we extended Recommendation 8 (Line 620-622) to include a note about documenting and controlling pH as well as a paragraph in the Experimental Execution

section to provide background for this recommendation (Line 453-460).

Line 681: missing parentheses closure

Corrected.
