# Supplementary material for: Standardizing deoxygenation studies in coral biology
Source: Commun Biol. 2026 Oct 19;9:1367. doi: 10.1038/s42003-026-11025-x (PMC13680498; doi:10.1038/s42003-026-11025-x)
Supplement: Supplementary file 3 [file 42003_2026_11025_MOESM2_ESM.pdf]

Supplemental Material: Standardizing deoxygenation studies in coral biology

Wyatt C. Million[1] (ORCID No. 0000-0002-0554-7633), Rachel Alderdice[2] (0000-0002-4234-7673), Nicole J. Dilernia[1] (0000-0002-0872-7402), Michael Kühl[3] (0000-0002-1792-4790), Cesar O. Pacherres[3] (0000-0001-7562-0253), Mathieu Pernice[1] (0000-0002-3431-2104), Carly J. Randall[4] (0000-0001-8112-3552), Alexandra Skeer[1], David J. Suggett[1,5], Christian R. Voolstra[2] (0000-0003-4555-3795), Emma F. Camp[1] (0000-0003-1962-1336)

[1] Climate Change Cluster, University of Technology Sydney, Ultimo, NSW, Australia
[2] Department of Biology, University of Konstanz, Konstanz, Germany
[3] Marine Biological Section, Department of Biology, University of Copenhagen, Helsingør, Denmark
[4] Australian Institute of Marine Science, Townsville, QLD, Australia
[5] KAUST Coral Restoration Initiative (KCRI) and Division of Biological and Environmental Science and Engineering (BESE), King Abdullah University of Science and Technology, Thuwal, Saudi Arabia

Corresponding author: Wyatt C. Million, wyatt.million@uts.edu.au

Keywords: marine, deoxygenation, hypoxia, coral, methods

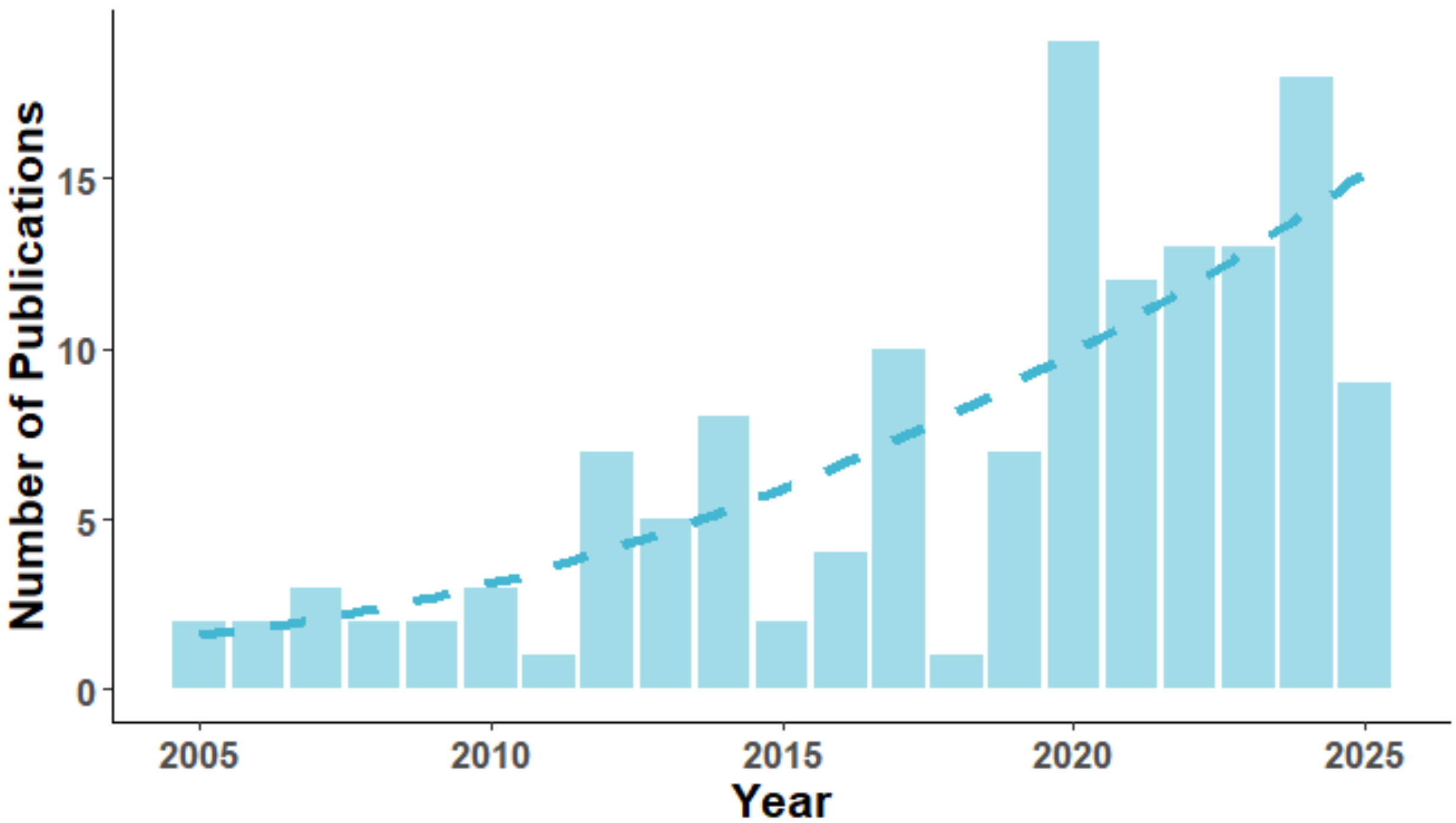

Figure S1: Number of peer-reviewed publications in coral deoxygenation and hypoxia each year over the last two decades. A Web of Science search using topic codes "coral" AND "deoxygenation" OR "hypoxia" OR "hypoxic" produced 365 publications. This list was manually filtered to remove duplicates and articles focusing on hypoxia in non-scleractinian organisms (e.g., studies in "coral-reef fish"). Articles were counted if they included *in situ* or *ex situ* investigation of the coral holobiont, coral biotic interactions (e.g., coral-macroalgae O_2 competition), or coral reef ecosystems. The final list of 143 publications highlights the growing consideration of low O_2 as an important stress factor in scleractinian coral. Lists of filtered and unfiltered Web of Science search results and search criteria can be found in the Supplementary Data.

Table S1: Sources of respirometry data used to make Figure 2.

Data	Source	Available at
Porties lutea<br>- Closed system respirometry data<br>- replicate #3<br>- Time vs. DO	Hughes *et al.* 2022 https://doi.org/10.1016/j.marpolbul.2022.113722	https://github.com/wyattmillion/CoralDeoxygenationPerspective
Hydnophora rigida<br>- Closed system respirometry data<br>- replicate #3<br>- Time vs. DO	Hughes *et al.* 2022 https://doi.org/10.1016/j.marpolbul.2022.113722	https://github.com/wyattmillion/CoralDeoxygenationPerspective

Table S2: Suggested checklist for reporting of coral deoxygenation methodology. Each item, if reported with the suggested minimum standards, would have sufficient context to be useful for cross-study comparisons.

**Data type/variable**	**Suggested minimum standards of reporting**
Aerobic threshold	Expressed in O_2 concentration (e.g., mg O_2 L^{-1} or µmol O_2 L^{-1}); type of respirometry used (closed vs. intermittent flow); length of incubations; type of analysis used to determine threshold
Sublethal/lethal threshold	Trait and degree of change used to determine threshold; O_2 concentration and duration of exposure to that condition (e.g. 50% reduction in F_v/F_m occurred at 1 mg O_2 L^{-1} after 12 hours)
Tolerance-modulating Phenotype	O_2 concentration and duration of exposure that triggered or preceded the observed phenotypic change
O_2 sensors and calibration	Temperature, salinity, pressure, and flow during calibration and throughout experiment
Closed system respirometry	Chamber volume; biomass-to-water volume ratio; sample surface area; rate of background respiration; temperature, salinity, pressure under which DO is measured, flow rate within closed system
Manual generation of deoxygenated water	Type of gas used (N_2, Air, CO_2, O_2); way in which gas was introduced to water; water turnover rate (L min^{-1}) and velocity of circulation within experimental tanks (m s^{-1}); pH
Intensity of deoxygenation	Expressed in absolute value of O_2 concentration (mg O_2 L^{-1} or µmol O_2 L^{-1}) or % air saturation as a function of temperature, salinity, and pressure; relationship to DO of source habitat and/or known aerobic, sublethal, or lethal threshold for the individual or species if possible; minimum and maximum DO and schedule for treatments if using diel fluctuations
Duration of deoxygenation	Length of experiment in days or hours; length of deoxygenation exposure per 24 hours; if using diel fluctuations, length of daytime reoxygenation and ramp up/ramp down period
Ramp rate of deoxygenation/reoxygenation	Magnitude of O_2 change/duration of change (e.g. mg O_2 L^{-1} $hour^{-1}$)
Sampling scheme	Timing of sampling relative to time of day, duration of deoxygenation exposure, and relative to diel cycle of O_2; location of sampling relative to original colony morphology

	(if possible) or final coral morphology post fragmentation; sample size
Experimental acclimation	Length of acclimation period prior to start of deoxygenation treatments; length of acclimation period immediately before respirometry after corals are moved to incubation chambers
Experimental feeding	Feeding volume, composition, frequency, and delivery method and if applicable; feeding regime prior to respirometry assays
Maintenance regime	Natural vs artificial seawater, filtration level, frequency/volume of water changes in recirculating system
Data analysis	The calculations or statistic programs used to derive critical phenotypes; the units of [DO] used to derive critical phenotypes; standardizations applied to VO_2 prior to calculation of P_{crit}, α-P_{crit}, or T_{pos}
Data accessibility	Access to [DO] vs time data from closed system respirometry or VO_2 vs. [DO] data from intermittent flow respirometry (rather than derived critical phenotypes only)

References:

Hughes, D. J. *et al.* Widespread oxyregulation in tropical corals under hypoxia. *Mar. Pollut. Bull.* **179**, 113722 (2022).
