# Supplementary material for: Standardizing deoxygenation studies in coral biology
Source: Commun Biol. 2026 Oct 19;9:1367. doi: 10.1038/s42003-026-11025-x (PMC13680498; doi:10.1038/s42003-026-11025-x)
Supplement: Supplementary file 4 [file 42003_2026_11025_MOESM3_ESM.pdf]

Description of Additional Supplementary Files

File name: Supplementary Data 1

Description: Hypoxia Thresholds Summary file that contains collected metadata used in Figure 4

File name: Supplementary Data 2

Description: Source data for Figure 3 which is a list of deoxygenation studies and the phenotypes each measure.

File name: Supplementary Data 3

Description: Source data for Figure 2 composed of the raw HRC data for the H. rigida coral.

File name: Supplementary Data 4

Description: Source data for Figure 2 composed of the raw HRC data for the P. lutea coral.

File name: Supplementary Data 5

Description: Results of Web of Science Search for coral deoxygenation/hypoxia studies with noncoral based studies manually removed

File name: Supplementary Data 6

Description: Source data for Supplementary Figure 1 that listed the number of coral deoxygenation studies that were published in each year based on Web of Science search
